# A new multi-residue method for PFAS analysis in wastewater for environmental and public health risk assessment

**DOI:** 10.1007/s00216-026-06421-5

**Published:** 2026-03-12

**Authors:** Dalia Elabbadi, Harry Elliss, Megan Robertson, John Bagnall, Barbara Kasprzyk-Hordern

**Affiliations:** 1 Department of Chemistry, University of Bath, Claverton Down, Bath, BA2 7AY UK; 2 Centre of Excellence in Water-Based Early-Warning Systems for Health Protection, University of Bath, Claverton Down, Bath, BA2 7AY UK; 3 Institute of Sustainability and Climate Change, University of Bath, Claverton Down, Bath, BA2 7AY UK; 4 Wessex Water Service Ltd, Claverton Down, Bath, BA2 7WW UK

**Keywords:** Per-/polyfluorinated alkyl substances, Wastewater, LC-MS/MS, PFAS adsorbance

## Abstract

This work focuses on the detection and quantification of per-/polyfluorinated alkyl substances (PFAS) in influent wastewater, using wastewater-based epidemiology via a new multi-residue ultra performance liquid chromatography-tandem mass spectrometry (UPLC-MS/MS) method. This covers a range of PFAS classes including perfluoroalkyl carboxylic acids (e.g. PFHxA), perfluoroalkyl sulfonic acids (e.g. PFBS), precursors (e.g. EtFOSAA), perfluoroalkyl phosphonic (e.g. PFOPA) and phosphinic acids (e.g. PFPiA), and novel PFAS (e.g. Gen X). Weak-anion exchange (WAX) cartridges and hydrophilic-lipophilic balance (HLB) solid-phase extraction cartridges for sample preparation were compared, with higher intensities and recoveries seen for different compounds for both cartridges. Method detection limits ranged from 0.03 (PFBS) to 756.9 ng L^−1^ (MeFOSA). Average method accuracy was 95% with a precision of 17.9%. Elution solvents on HLB cartridges were tested (MeOH, ACN, and IPA), with MeOH found to work sufficiently for the analytes in this method. Some PFAS were found to adsorb to silanised glassware and HDPE material during sample preparation. The method was applied to influent wastewater samples from the South-West of England across three days to assess daily load and potential population exposure. Daily loads ranged from 1935.4 ± 146.9 (PFNA) to 45,368.9 ± 24,983.6 mg day^−1^ (8:2 diPAP), and population-normalised daily load ranged from 2.5 ± 0.2 to 55.2 ± 30.4 µg day^−1^ 1000 inh^−1^ for those same compounds. Risk from human exposure and acute risk to the environment were assessed and deemed to be low; however, risk can be difficult to assess due to more factors needing to be considered.

**Supplementary Information:**

The online version contains supplementary material available at 10.1007/s00216-026-06421-5.

## Introduction

Per-/polyfluorinated alkyl substances (PFAS) have been gaining global attention due to their persistence in organisms [1, 2], aquatic environments [3, 4], and terrestrial environments [5, 6]. They also have bioaccumulative properties [7] and are known to be bioamplified along a food chain [8], which is another threat to humans and animals. This is a major public health concern given their link to thyroid, liver, kidney, and reproductive issues. They have also been linked to certain cancers [9, 10]. As a result, the use of PFOS, PFOA, and PFHxS has been restricted by the persistent organic pollutants (POPs) Stockholm convention [11]. Longer-chain PFAS have been voluntarily phased out by industry [12].

The Organisation for Economic Co-operation and Development (OECD) and the Environment Agency adopted the definition of PFAS that states that they are any chemical with at least one fully (per-) fluorinated methyl (–CF_3_) or methylene (–CF_2_–) group [13, 14]. Perfluoroalkyl substances describe those where all backbone carbons have been replaced with fluorine, whereas polyfluorinated compounds describe those where all hydrogens on at least one carbon have been replaced with fluorine [15]. They all structurally have a fluorinated carbon chain attached to a functional group, such as a carboxylate or sulfonate group [16]. The presence of these strong carbon–fluorine bonds means that PFAS are highly physically and thermally stable [17] and do not degrade easily [18]. They also have non-stick properties, giving them use as surfactants and as non-stick surfaces, both domestically and industrially [19].

Some of the major classes that have been investigated and used extensively include perfluoroalkyl carboxylic acids (PFCA), perfluoroalkyl sulfonic acids (PFSA), perfluoroalkyl phosphinic acids (PFPiA), perfluoroalkyl phosphonic acids (PFPA), and perfluoroalkyl ether carboxylic or sulfonic acids (PFECA and PFESA). Fluoropolymers and fluoropolyethers are also widely used. PFAS also have precursors, which are grouped into classes such as n:2 fluorotelomer-based substances (n:2 FTS), perfluoroalkyl ether-based substances, perfluoroalkyl alcohols, and hydrofluorocarbons (HFC) [15, 20, 21]. PFAS can be referred to by multiple names; for example, EtFOSA is also known as sulfluramid, and HFPO-DA is also commonly known as Gen X; therefore, CAS numbers are crucial to avoid confusion across studies [15, 22].

There are several ways in which both humans and the environment are exposed to PFAS. Industrial waste is a major source of PFAS due to their widespread and extensive use and subsequent difficulty to remove [23, 24]. Furthermore, PFAS are found in high concentrations in aqueous film-forming foam (AFFF), causing PFAS to enter soil and groundwater systems [25], and have been detected in the serum of firefighters [26]. Domestic use of appliances is also a source of PFAS exposure, given the extensive historical use of PFAS in everyday items such as non-stick cookware, waterproof clothing, and personal care products [23, 27]. This leads to ingestion and dermal absorption of these chemicals [28], and run-off from these can enter the environment. There is a strong interconnectedness between all of these sources; for example, contaminated water is often used for agricultural irrigation [29], further continuing the cycle of PFAS sources in the environment. Therefore, a One Health framework is needed to address this issue.

In light of regulatory restrictions on PFAS use, novel replacements for traditional PFAS are seeing increased use, such as 6:2 FTS, which can also act as a precursor to the compounds it was introduced to replace [30]. Gen X and ADONA act as replacements for PFOA [31, 32], and F-53B (also known as 9ClPF_3_ONS) is being used to replace PFOS [33]. The difference that these novel replacements have is an ether functional group structurally, sometimes the addition of a heteroatom, and a shorter carbon chain [32]. These compounds have been detected and quantified in a range of aqueous matrices [10, 34]. As concern over the long-lasting presence of PFAS grows, it becomes more crucial to monitor their levels and presence across environments and communities. Wastewater-based epidemiology (WBE) presents the opportunity to do so, given that wastewater reflects the chemicals used by a community and what is subsequently released into the environment. It is a novel analytical tool that is anonymous and can quantify levels of compounds in near real time [35, 36]. It has traditionally been used to monitor licit and illicit drug consumption [37, 38], as well as antibiotic use [39] and oxidative stress [40]. It has also been used successfully to evaluate levels of exposure to chemicals of emerging concern, from bisphenols [41] to pesticides [42, 43]. 

The main limitation for using WBE to understand levels of PFAS exposure from a public health standpoint is the lack of a characteristic metabolite for these persistent compounds [44]. There have been numerous studies detecting and quantifying PFAS in wastewater, in Australia [45, 46], Canada [47], Belgium [48], Sweden [49], and Korea [50], using both targeted and untargeted methods.

We present a targeted analytical method for 35 PFAS compounds spanning multiple classes, with 4 isotopically labelled PFAS standards and 4 isotopically labelled non-PFAS standards. This method focuses on the most hazardous and prevalent compounds to allow for a deeper understanding of public and environmental exposure to these substances, as well as low limits of detection for trace analysis.

## Method

### Chemicals/reagents

Analytical standards were purchased from Sigma-Aldrich (Gillingham, UK) and LGC Standards (Teddington, UK). These were purchased in either solid or liquid form and dissolved in methanol to make 1.0 mg mL^−1^ solutions. These were then used to make analyte mixes of varying concentrations for spiking. Isotopically labelled standards (ISTDs) were purchased from CK Isotopes LTD (Leicestershire) in methanolic solution, at 50 µg mL^−1^. All solutions were stored in the freezer at −20 °C to ensure stability. More information on standards purchasing can be found in S1. LC–MS grade solvents, Whatman GF/F 0.7-µm filters, and ammonium fluoride were purchased from Sigma-Aldrich. SPE cartridges (Oasis HLB, 60 mg, 3 mL, and Oasis WAX, 60 mg, 3 mL) and LC polypropylene vials were purchased from Waters (Manchester, UK). HDPE bottles (125 mL) were purchased from Fisher Scientific.

### Target list selection

The target list consists of 35 PFAS. They were selected to include as many different classes of PFAS as possible, both from use as well as from physicochemical properties. As PFAS span thousands of chemicals [51], it can be difficult to capture the most important ones. Therefore, this target list selection was based upon compounds regularly reported on in literature, compounds categorised by HBM4EU[52] (A Horizon funded Human Biomonitoring (HBM) project which later became PARC[53]) [53–55] and novel PFAS that may not have been extensively reported on. The target list includes perfluoroalkyl carboxylic acids and their precursors, perfluoroalkyl sulfonic acids and their precursors, perfluorophosphonic acids, perfluorophosphinic acids, and novel/emerging ethers. The full target list can be seen in S2.

### Sample preparation

A total of 50 mL of wastewater was spiked with 100 ng mL^−1^ of ISTD. These were then spiked with an analyte mix at 4 different concentrations: 5, 20, 200, and 500 ng mL^−1^, with one unspiked sample kept, capturing concentrations of PFAS already present in the sample, or PFAS introduced through lab work. Analysis was performed in triplicate for both WAX and HLB cartridges. For samples undergoing WAX SPE, samples were trialled fully either left at the native pH range (WAX A) or adjusted to pH 2 using 2M HCl (WAX B). Samples were left to equilibrate the standards and internal standards with the matrix for half an hour. The samples were then filtered using a vacuum filtration device with a 0.7 µm GF/F glass-fibre filter.

#### HLB cartridges

Cartridges were conditioned with 2 mL MeOH followed by 2 mL H_2_O under gravity. Samples were loaded onto cartridges at a flow rate of 5 mL min^−1^, then dried under vacuum for 30–60 min. Samples were eluted using 4 mL MeOH under gravity and dried under nitrogen using a Turbovap LV concentration workstation (Caliper, UK, < 5 psi, 40 °C). Samples were then reconstituted using 100 µL of MeOH, then 400 µL H_2_O, and transferred into LC polypropylene vials.

#### WAX cartridges

Cartridges were conditioned with 2 mL 0.5% NH_4_OH in MeOH (v/v), followed by 2 mL MeOH, followed by 2 mL H_2_O, all under gravity. Once loaded with sample, cartridges were washed with 25 mM acetate buffer (pH 4) under vacuum. Samples were eluted using 3 mL MeOH (which was discarded), followed by 3 mL 0.5% NH_4_OH in MeOH (v/v). Following extraction, samples were dried under nitrogen using a Turbovap LV concentration workstation (Caliper, UK, < 5 psi, 40 °C). Samples were then reconstituted using 100 µL of MeOH, then 400 µL H_2_O, and transferred into LC polypropylene vials.

S3 shows the sample preparation on both cartridges in schematic form.

#### Elution solvent comparison

For the elution solvent comparison, SPE was carried out as outlined above on HLB cartridges, at two concentration levels: 20 ng mL^−1^ and 100 ng mL^−1^, as well as one blank (unspiked) sample to calculate native concentration. The changes to the elution step were as shown in Table 1. These were then dried and reconstituted as above.

**Table 1:** Combinations of elution solvent assessed in this study

	IPA	ACN
Elution 1	4 mL MeOH	4 mL MeOH
Elution 2	2 mL MeOH then 2 mL IPA	2 mL MeOH then 2 mL IPA
Elution 3	4 mL IPA	4 mL IPA

### Adsorbance to HDPE material

Analytes were spiked at 20 ng mL^−1^ and 200 ng mL^−1^ into 400 µL LC–MS water in a HDPE bottle, alongside 100 ng mL^−1^ ISTD. The sample was left for 40 min at room temperature to represent the approximate length of time analytes may spend in a HDPE bottle during sample preparation. This was then transferred straight into a vial ready for LCMS analysis.

### Liquid chromatography

Chromatography was carried out using a Waters Acquity UPLC system (Waters, Manchester, UK) with a BEH C18 column (150 × 1.0 mm, 1.7 µm particle size) (Waters, Manchester, UK) with a 0.2-µm, 2.1-mm in-line column filter. The column temperature was 25 °C. The injection volume was 20 µL.

Two mobile phases were used: Mobile phase A was 80:20 H_2_O:MeOH + 1 mM NH_4_F, and mobile phase B was 5:95 H_2_O:MeOH + 1 mM NH_4_F. Strong and weak needle washes consisted of 1:1:1:1 IPA:ACN:H_2_O:MeOH and 95:5 H_2_O:MeOH, respectively. The gradients used were as follows: 0–0.5 min 0%B, 0.5–2.5 min 60%B, 2.5–8.0 min 100%B, 8.0–14.0 min 100%B, 14.0–14.1 min 100%A, 14.1–22.5 min 100%A. This chromatographic method was adapted from a previously published method [56].

### Mass spectrometry

A Xevo TQD Triple Quadrupole Mass Spectrometer (Waters, Manchester, UK) equipped with an electrospray ionisation source (ESI) and operated in the negative ionisation mode. Nitrogen was used as a desolvation and nebulising gas, and argon was used as the collision gas. The MS settings were as follows: capillary voltage of 3.0 kV, desolvation temperature of 400 °C, source temperature of 150 °C, cone gas flow of 100 L h^−1^, and desolvation gas flow of 700 L h^−1^.

Samples were analysed in multiple reaction monitoring (MRM) mode, with the two most abundant precursors to product transitions, after manual tuning, used. The mass spectrometer was operated with Waters MassLynx 4.2, and TargetLynx was used for data processing.

### Instrument performance

For quantification, calibration curves were prepared containing analytical standards and internal standards in 80:20 H_2_O:MeOH. These solutions ranged from 0.01 to 1000 ng mL^−1^ over 19 calibration points to assess instrumental performance, with triplicate injections. These were used to assign internal standards and to assess their linearity against each target. The instrumental quantification limit (IQL) was taken as the lowest measured concentration at which the most injections revealed a quantification trace with a S/N of ≥ 10, and a confirmation trace with a S/N of ≥ 3.3. Please see the following papers for further details [57–59].

The IDL was calculated using Eq. 1:

1 $$IDL=\frac{IQL}{10\times3.3}$$

Mobile phase QCs were prepared and injected with every batch of samples. They consisted of analytical standards and internal standards in 80:20 H_2_O:MeOH at 4 concentrations: 5, 20, 200, and 500 ng mL^−1^, as PFAS are often detected at trace levels. They were replaced with fresh solutions daily. These were used to calculate interday and intraday accuracy and precision according to Eqs. 2 and 3,

2 $$Accuracy\;\left(\%\right)\;=\frac{Cs,\;exp}{Cs,\;theo}\times100$$

3 $$Precision\;\left(RSD\right)=\sqrt{\frac1{N-1}\times{\textstyle\sum_{i=1}^N}\left({Ci,}_{exp}-C_{exp}\right)^2}$$

where *C*_s_,_exp_ is the concentration of the sample derived experimentally, and *C*_s_,_theo_ is the concentration that was spiked in.

### Method performance

Method recovery was assessed using influent wastewater samples (matrix QCs) spiked at 5, 20, 200, and 500 ng mL^−1^ and calculated using Eq. 4:

4 $$Recovery\;\left(\%\right)\;=\frac{C_{ss}-C_{us}}{C_{theo}}\times100$$

where *C*_ss_ is the experimentally derived concentration, and *C*_us_ is the experimentally derived concentration of the unspiked sample. Matrix QC samples were injected regularly throughout the run.

The method quantification limit (MQL) was calculated using the IQL according to Eq. 5:

5 $$MQL=\frac{IQL\times100}{Recovery\;\times Cf}$$

Cf is the conversion factor for recovery of a certain matrix, which for influent wastewater is 100.

The method detection limit (MDL) was calculated using the same Eq. [5], replacing ‘IQL’ with ‘IDL’.

Recovery, accuracy, and precision data can be found in the results section in Table 4 (instrument) and Table 5 (method).

### WBE calculations

The concentrations were used to calculate daily mass loads (DL), expressed in mg day^−1^ allowing for an understanding of the amount of PFAS entering the WWTP per day. This was calculated using Eq. 6:

6 $$DL\;\left(mg\;day^{-1}\right)=C\times V$$

where *C* is the concentration in mg L^−1^, and *V* is the flow rate entering the WWTP in L day^−1^. To allow for direct comparison between sites, the population-normalised daily load (PNDL), expressed in mg day^−1^ 1000 inh^−1^, was calculated using Eq. 7:

7 $$PNDL\;\left(mg\;day^{-1}\;1000inh^{-1}\right)=\frac{DL}P\times1000$$

where *P* is the number of inhabitants served by the WWTP.

Flow and population data can be found in S4.

#### Environmental risk assessment

An environmental risk assessment was carried out for untreated influent wastewater, using the risk quotient method, according to Eq. 8:

8 $$Risk\;Quotient\;\left(RQ\right)\;=\frac{MEC}{lowest\;PNEC}$$

where MEC is the measured environmental concentration, and PNEC is the predicted no-effect concentration in freshwater, taken from the Norman ecotoxicology database, and is shown in Table 2 [60].

Risk quotients were evaluated as low risk (RQ < 0.1), medium risk (0.1 ≤ RQ < 1), and high risk (RQ ≥ 1) [61].

**Table 2:** Lowest PNECs in freshwater recorded for PFAS that were quantifiable in this study. Organisms are included

	PFBS	PFOA	PFNA	PFDA	8:2 diPAP	PFOPA	PFDPA
PNEC value (ng L^−1^)	372,000	178	1000	165.37	73.39	630	290
Organism (taxonomic group, scientific name)	Not recorded	Insect, *Chironomus riparius*	Fish, *Pimephales Promelas*	Fish, *Pimephales Promelas*	Fish, *Pimephales Promelas*	Fish, *Pimephales Promelas*	Fish, *Pimephales Promelas*
Chronic or Acute	Not recorded	Chronic	Acute	Acute	Acute	Acute	Acute

## Results and discussion

### Liquid chromatography-tandem mass spectrometry

As PFAS are acidic compounds, they were all analysed using ESI− mode, with [M-H]^−^ selected as the parent ion. This method was developed by building on an existing method in the group [56, 57, 62]. Figure S5 shows a TIC for the PFAS analysed in this method, to show their chromatographic separation and retention time windows. Table 3 shows the MRM transitions for the target list of compounds ordered by PFAS class, alongside their assigned internal standards. Most compounds have two MRM transitions for accurate identification and quantification, with the most abundant transition used for quantification and the less abundant transition used for confirmation. The following compounds, PFBA, PFOPA, PFDPA, and 8:8 PFPiA, did not have a second transition. This aligns with other studies looking at these compounds where a second transition could also not be found and could be structure-related [48, 63].

**Table 3:** MRM transitions for the target list of PFAS in this method

**Analyte class**	**Analyte**	**MRM 1**	**MRM 1 CV/CE**	**MRM 2**	**MRM 2 CV/CE**	**ISTD**
PFSAs	PFBS	298.90 > 98.9	40/27	298.90 > 79.9	40/28	PFBS ^13^C_4_
	PFHxS	398.95 > 79.90	55/37	398.95 > 98.9	55/32	PFBS ^13^C_4_
	PFHpS	448.80 > 168.95	50/31	448.80 > 229.90	50/22	6:2 FTS ^13^C_2_ D_4_
	PFOS	498.80 > 98.9	41/41	498.80 > 169	41/34	6:2 FTS ^13^C_2_ D_4_
	PFNS	548.90 > 98.9	50/45	548.90 > 118.90	50/45	6:2 FTS ^13^C_2_ D_4_
Precursors to PFSAs	PFOSA	497.80 > 77.9	44/43	497.80 > 168.9	44/26	PFBS ^13^C_4_
	n-MeFOSA	511.90 > 168.9	45/26	511.90 > 218.9	45/25	6:2 FTS ^13^C_2_ D_4_
	n-EtFOSA	525.80 > 169.0	39/28	525.80 > 218.90	39/24	6:2 FTS ^13^C_2_ D_4_
	n-MeFOSAA	569.90 > 418.95	30/18	569.90 > 482.90	30/15	Ibuprofen D_3_
	n-EtFOSAA	583.80 > 218.90	29/26	583.80 > 418.90	29/19	Ketoprofen D_3_
PFCAs	PFBA	212.90 > 168.90	17/8	-	-	PFBA ^13^C_4_
	PFPeA	262.90 > 68.90	15/40	262.90 > 218.90	15/7	Ibuprofen D_3_
	PFHxA	312.9 > 118.9	12/20	312.9 > 268.95	12/9	Ibuprofen D_3_
	PFHpA	362.90 > 168.90	16/17	362.90 > 318.90	16/9	Ibuprofen D_3_
	PFOA	412.90 > 168.90	20/17	412.90 > 368.90	20/9	PFOA ^13^C_8_
	PFNA	462.8 > 218.95	17/15	462.80 > 418.90	17/9	PFOA ^13^C_8_
	PFDA	512.80 > 218.90	13/15	512.80 > 468.90	13/11	PFOA ^13^C_8_
	PFUnDA	562.90 > 269.0	15/18	562.90 > 518.90	15/10	6:2 FTS ^13^C_2_ D_4_
	PFDoDA	612.80 > 168.90	19/25	612.80 > 569.00	19/13	PFOA ^13^C_8_
	PFTrDA	662.80 > 168.9	18/26	662.80 > 218.90	18/24	PFOA ^13^C_8_
	PFTeDA	712.90 > 168.9	17/28	712.9 > 668.9	17/13	Ketoprofen D_3_
Precursors to PFCAs	6:2 FTS	426.80 > 386.90	38/26	426.80 > 407.0	38/21	6:2 FTS ^13^C_2_ D_4_
	8:2 FTS	526.95 > 80.95	45/42	526.95 > 506.95	45/26	6:2 FTS ^13^C_2_ D_4_
	5:3 FTC	340.9 > 216.95	23/24	340.9 > 237.0	23/16	PFBS ^13^C_4_
	6:2 diPAP	788.9 > 96.9	40/30	788.90 > 442.90	40/20	Methyl Paraben ^13^C_6_
	8:2 diPAP	988.85 > 96.9	44/35	988.85 > 542.9	44/23	Methyl Paraben ^13^C_6_
Phosphonic/phosphinic acids	PFHxPA	398.9 > 79.9	42/40	398.90 > 118.90	42/34	PFOA ^13^C_8_
	PFOPA	498.9 > 78.90	45/31	-	-	6:2 FTS ^13^C_2_ D_4_
	PFDPA	598.90 > 78.9	52/40	-	-	6:2 FTS ^13^C_2_ D_4_
	6:6 PFPiA	700.85 > 100.9	55/61	700.85 > 400.9	55/45	PFOA ^13^C_8_
	8:8 PFPiA	900.8 > 500.8	72/61	-	-	PFOA ^13^C_8_
	8:2 monoPAP	542.90 > 96.90	30/18	542.90 > 522.90	30/13	6:2 FTS ^13^C_2_ D_4_
Novel emerging PFAS	Gen X	284.80 > 168.90	16/8	284.80 > 184.90	16/16	PFBS ^13^C_4_
	ADONA	376.90 > 84.90	16/26	376.90 > 250.90	16/10	PFBS ^13^C_4_
	9ClPF_3_ONS	530.80 > 98.90	42/30	530.80 > 350.90	42/26	Acetaminophen D_4_
Non-PFAS ISTDs	PFBS ^13^C_4_	302.9 > 79.9	40/29	-	-	-
	PFBA ^13^C_4_	216.90 > 171.90	17/8	-	-	-
	PFOA ^13^C_8_	420.9 > 375.90	17/9	-	-	-
	6:2 FTS ^13^C_2_ D_4_	432.90 > 411.90	40/22	-	-	-
	Acetaminophen D_4_	154.00 > 111.00	38/19	-	-	-
	Methyl Paraben ^13^C_6_	156.90 > 97.90	30/20	-	-	-
	Ketoprofen D_3_	256.00 > 212.00	15/7	-	-	-
	Ibuprofen D_3_	208.00 > 164.00	20/6	-	-	-

### Comparison of SPE sorbent

**Fig. 1:**
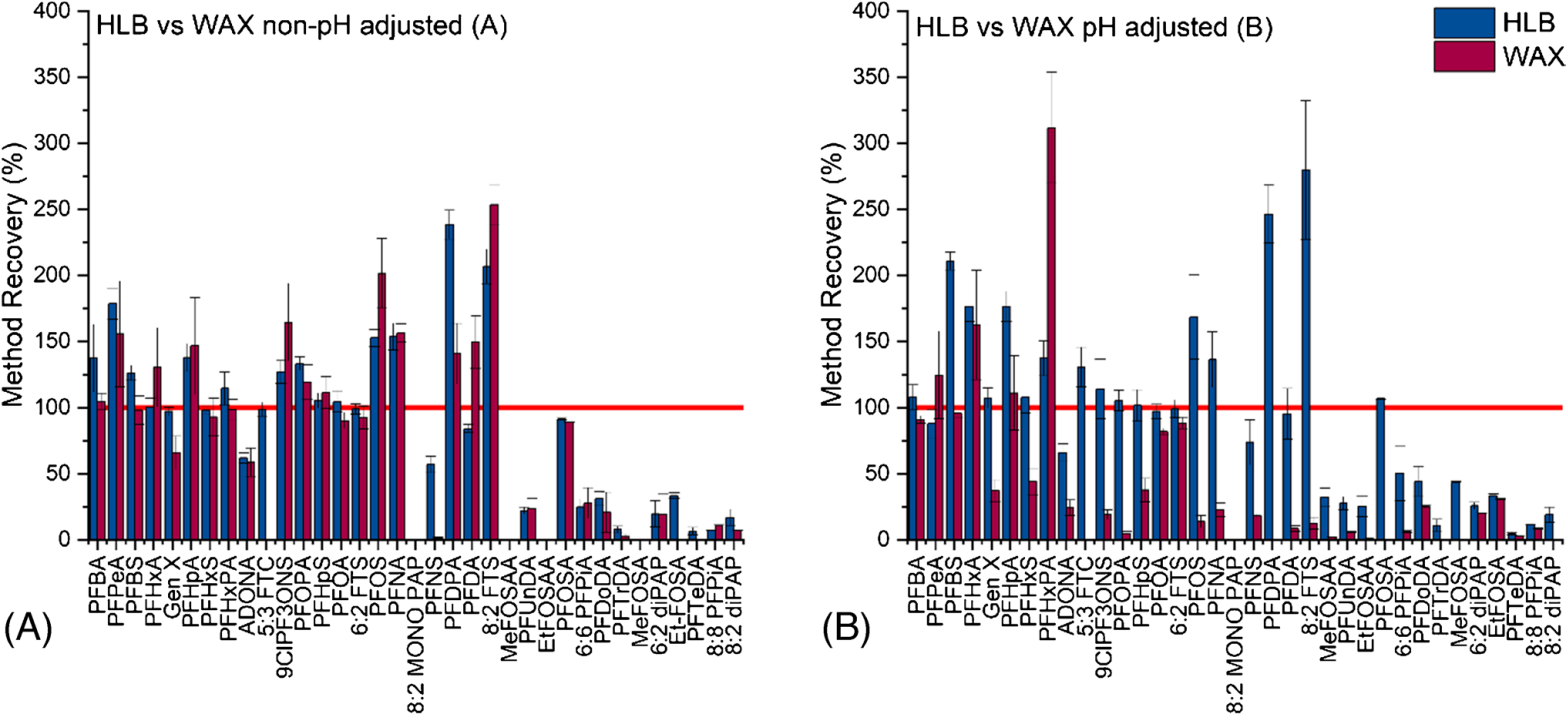
Method recovery for HLB vs WAX A (non-pH adjusted) (**A**) and HLB vs WAX B (pH < 2) (**B**) at 200 ng mL^−1^ (except PFDoDA, PFTrDA, 8:2 diPAP, PFHxPA, 6:6 PFPiA, and 8:2 monoPAP, at 20 ng mL^−1^). The red line shows 100% method recovery on the *y*-axis

HLB cartridges were compared against WAX cartridges (both with not pH adjusted samples (A) and pH < 2 adjusted samples (B)). To compare the performance of the two cartridges using this method, method recovery and signal intensity were used as indicators. Firstly, method recovery for the three SPE methods is shown in Fig. 1 and S6, with compounds ordered by retention time. Different wastewater samples were used for the evaluation of WAX A and WAX B; therefore, recovery on HLB was repeated for both sets of influent wastewater as a control. Comparison of method recovery was assessed at the 200 ng mL^−1^ spiked level for all compounds except for PFDoDA, PFTrDA, 8:2 diPAP, PFHxPA, 6:6 PFPiA, and 8:2 monoPAP, as this concentration was out of their calibration range, so recovery was assessed at 20 ng mL^−1^ instead.

Comparing HLB with WAX (A), the PFAS with a better method recovery (i.e. closer to 100%) on WAX cartridges were PFBA (by 32.8%), PFPeA (by 22.6%), PFBS (by 28.1%), PFHxPA (by 15.9%), PFOPA (by 14.0%), and PFDPA (by 97.6%). The two compounds PFUnDA and 6:6 PFPiA showed better recovery on WAX, by less than 4%, and therefore had comparable recoveries. Comparing HLB with WAX (B), the only compound with a higher method recovery on WAX cartridges was PFBS. PFBA could not be quantified at 5 ng mL^−1^ on HLB cartridges; however, this was possible on WAX A and B cartridges. 5:3 FTC had negative recovery values for WAX A and B cartridges due to low peak areas. This is likely due to how telomerisation reduces acidity, with 5:3 FTC being 10^4^ times less acidic than its perfluorinated analogue PFOA [64]. Generally, as retention time increased, suitability with WAX cartridges also decreased.

Both HLB [50, 65, 66] and WAX [46, 47] cartridges have been widely used in the literature for PFAS sample preparation. It has been suggested that HLB cartridges can enhance the detection of a wider range of PFAS in wastewater than WAX cartridges [67], and that SPE on ultrapure water showed better recoveries on HLB cartridges than WAX [68]. A study looking at HLB and WAX cartridges over three pH levels with seawater as the matrix found better recoveries on HLB cartridges for PFCAs, and better recoveries with WAX cartridges for PFSAs [69]. This makes sense given PFSA’s lower pKa (S2). Another study found high recoveries for a range of PFAS using HLB cartridges, however even better recoveries on WAX for certain compounds, especially short-chain PFAS, which aligns with these results [70]. This suggests that both HLB and WAX cartridges could be used together for optimum PFAS analysis in wastewater, with WAX cartridges potentially enhancing recovery of shorter-chain PFAS at trace levels and HLB cartridges allowing capture of longer-chain PFAS. This method is part of a wider method screening for multi-class compounds with wide polarity ranges, hence our selection of less selective HLB cartridges moving forward.

### Instrument performance

Table 4 shows the parameters for instrumental performance in the mobile phase, ordered by PFAS class. These parameters were retention time, linearity, ion ratio, IDL and IQL (to indicate sensitivity), and accuracy and precision (to indicate performance). Internal standards were assigned based on a few parameters, such as linearity of response, relative retention time, structural similarity, recovery, and comparable ionisation efficiency.

Retention times for the analytes were consistent across vials and injections, with little deviation. The retention times for the target list spanned from 6.99 ± 0.02 to 13.81 ± 0.01 min across a 22.5-min method. These generally matched well with the assigned internal standards, with relative retention times (RRT) ranging from 0.75 ± 0.00 to 1.97 ± 0.01. Linearity was assessed using calibration curves in the mobile phase, and over half of the compounds had an *R*^2^ value of 0.997, and all others were above 0.99, except for PFTrDA, PFOSA, and MeFOSA.

Values for the IDLs ranged from 0.0033 to 33 µg L^−1^, and values for the IQLs ranged from 0.01 to 100 µg L^−1^. The highest IQLs were seen for the class of sulfonic acid precursors, with PFOSA and MeFOSA at 100 µg L^−1^. Following this were PFHpS and EtFOSA at 50 µg L^−1^ and PFNS and 9ClPF_3_ONS at 25 µg L^−1^. The lowest quantification limits were found for PFBS, PFBA, PFTeDA, and 8:2 diPAP, at 0.01 µg L^−1^.

Variability in the ion ratio should ideally be within 40% for compound identification. Closer to the IQL, higher variability was seen for some compounds; therefore, ion ratios have been reported in two ranges: from 0 to 100 µg L^−1^ and from 100 to 1000 µg L^−1^ to give a clearer picture, as has been done previously [71]. Ion ratios were generally consistent from 100 to 1000 µg L^−1^ and within the acceptable range. The following compounds showed higher variability at the lower end of the calibration scale, closer to the IQL: PFHpS, PFPeA, PFHpA, 9ClPF_3_ONS, 8:2 monoPAP, 6:6 PFPiA, and 6:2 FTS.

Instrumental accuracy and precision were evaluated for all concentrations within the calibration range for each compound, over 4 concentrations: 5, 20, 200, and 500 ng mL^−1^. The compound was assumed fully quantitative for accuracy 50–120% and precision < 30%, semi-quantitative for accuracy 30–50% and precision 30–50%, and qualitative for accuracy and precision beyond these parameters, regardless of calibration curve linearity. The best accuracy and precision were seen for PFBS (98.7 ± 7%), PFHxS (97.1 ± 6.5%), and 6:2 FTS (102.0 ± 4.4%). Compounds with worse accuracy and precision were PFTrDA (41.3 ± 13.7%), PFTeDA (39.7 ± 11.7%), and 8:2 monoPAP (39.7 ± 24%). For some compounds, QC 5 was omitted during the calculation of accuracy and precision where high variability was seen across vials and injections, likely from being close to the quantification limit which can introduce analytical uncertainties. Interday and intraday accuracies and precision were overall similar without significant variation. Poor instrument performance and linearity were seen for the more hydrophobic compounds. This could be from a lack of matching ISTDs, or that they may be adsorbing to glass-fibre or plastic vials/lab equipment during standards stock preparation.

**Table 4:** Instrument performance parameters for PFAS in the mobile phase

	**Analytes**				**Absolute and relative retention times**		**Ion ratio**		**Calibration curve ranges and linearity**		**Instrumental Limits**		**Intraday**		**Interday**	
Analyte class	Analyte name	ISTD used		Quantification	RT (mins)	RRT (mins)	IQL–100 (µg L^−1^)	100–1000 (µg L^−1^)	Linear Range (µg L^−1^)	Linearity	IDL (µg L^−1^)	IQL (µg L^−1^)	Accuracy (%)	Precision (%)	Accuracy (%)	Precision (%)
PFSAs	PFBS		PFBS ^13^C_4_	Quantitative	7.80 ± 0.01	1.00 ± 0.00	0.52 ± 0.10	0.52 ± 0.01	IQL–1000	0.9997	0.0033	0.01	98.7	7.0	99.1	13.3
	PFHxS		PFBS ^13^C_4_	Quantitative	9.05 ± 0.02	1.16 ± 0.00	1.59 ± 0.26	1.63 ± 0.03	IQL–500	0.9995	0.033	0.1	97.1	6.5	97.3	5.8
	PFHpS*		6:2 FTS ^13^C_2_ D_4_	Semi-quantitative	9.70 ± 0.01	1.00 ± 0.00	66.36 ± 39.67	44.07 ± 10.62	IQL–600	0.9981	16.5	50	129.9	6.8	123.1	8.4
	PFOS*		6:2 FTS ^13^C_2_ D_4_	Semi-quantitative	10.30 ± 0.03	1.06 ± 0.00	6.22 ± 4.39	3.80 ± 0.37	IQL–400	0.9982	0.165	0.5	140.7	21.1	141.0	10.1
	PFNS		6:2 FTS ^13^C_2_ D_4_	Quantitative	10.88 ± 0.01	1.13 ± 0.00	9.48 ± 7.19	5.94 ± 1.45	IQL–600	0.9901	8.25	25	113.0	13.6	102.9	20.9
PFSA Precursors	PFOSA*		PFBS ^13^C_4_	Semi-quantitative	11.69 ± 0.09	1.50 ± 0.01	---	23.30 ± 10.70	IQL–800	0.9396	33	100	83.1	26.5	77.9	28.8
	n-MeFOSA*		6:2 FTS ^13^C_2_ D_4_	Semi-quantitative	12.62 ± 0.03	1.31 ± 0.00	---	1.62 ± 0.72	IQL–1000	0.9228	33	100	91.4	24.6	84.7	30.4
	n-EtFOSA		6:2 FTS ^13^C_2_ D_4_	Quantitative	13.02 ± 0.14	1.35 ± 0.01	2.62 ± 2.23	1.43 ± 0.20	IQL–400	0.9917	16.5	50	62.7	10.9	52.4	20.0
	n-MeFOSAA		Ibuprofen D_3_	Quantitative	11.22 ± 0.03	1.10 ± 0.00	3.39 ± 4.70	2.03 ± 0.10	IQL–500	0.9907	0.33	1	90.5	14.5	85.4	16.3
	n-EtFOSAA		Ketoprofen D_3_	Quantitative	11.50 ± 0.03	1.39 ± 0.01	3.90 ± 2.06	2.91 ± 0.17	IQL–600	0.9935	3.3	10	84.3	19.9	69.4	25.2
PFCAs	PFBA		PFBA ^13^C_4_	Quantitative	6.99 ± 0.02	0.99 ± 0.03	---	---	IQL–1000	0.9993	0.0033	0.01	84.6	21.6	87.9	20.2
	PFPeA		Ibuprofen D_3_	Quantitative	7.71 ± 0.02	0.75 ± 0.00	737.55 ± 1153.64	425.40 ± 80.43	IQL–250	0.9984	3.3	10	109.6	4.1	114.4	8.7
	PFHxA		Ibuprofen D_3_	Quantitative	8.39 ± 0.03	0.82 ± 0.00	14.03 ± 3.46	12.62 ± 0.41	IQL–200	0.998	0.165	0.5	90.2	25.7	102.1	30.3
	PFHpA		Ibuprofen D_3_	Quantitative	9.05 ± 0.03	0.88 ± 0.00	5.78 ± 8.26	4.04 ± 0.16	IQL–250	0.9985	0.0165	0.05	104.2	23.1	118.9	25.4
	PFOA		PFOA ^13^C_8_	Quantitative	9.70 ± 0.03	1.00 ± 0.00	3.51 ± 2.08	3.00 ± 0.10	IQL–500	0.9997	0.0165	0.05	87.3	5.4	88.8	5.1
	PFNA		PFOA ^13^C_8_	Quantitative	10.32 ± 0.02	1.06 ± 0.00	4.73 ± 0.97	4.21 ± 0.12	IQL–600	0.9910	0.33	1	119.0	27.9	118.9	28.8
	PFDA		PFOA ^13^C_8_	Quantitative	10.90 ± 0.02	1.12 ± 0.00	7.26 ± 3.98	5.38 ± 0.24	IQL–400	0.9989	0.165	0.5	102.7	27.6	100.0	22.1
	PFUnDA		6:2 FTS ^13^C_2_ D_4_	Quantitative	11.43 ± 0.02	1.18 ± 0.00	9.46 ± 5.30	7.23 ± 0.53	IQL–400	0.9954	3.3	10	116.3	23.3	114.6	24.9
	PFDoDA		PFOA ^13^C_8_	Quantitative	11.96 ± 0.06	1.23 ± 0.00	6.38 ± 2.89	5.30 ± 0.36	IQL–100	0.9948	1.65	5	94.8	19.7	93.4	28.7
	PFTrDA*		PFOA ^13^C_8_	Semi-quantitative	12.37 ± 0.01	1.28 ± 0.00	1.65 ± 0.36	1.61 ± 0.05	IQL–75	0.9861	0.165	0.5	41.3	13.7	35.0	17.0
	PFTeDA*		Ketoprofen D_3_	Semi-quantitative	12. 79 ± 0.02	1.55 ± 0.01	5.08 ± 1.17	5.29 ± 0.08	IQL–100	0.9899	0.0033	0.01	39.7	11.7	33.6	10.4
PFCA Precursors	6:2 FTS		6:2 FTS ^13^C_2_ D_4_	Quantitative	9.69 ± 0.03	1.00 ± 0.00	14.97 ± 18.69	9.30 ± 0.35	IQL–600	0.9999	0.165	0.5	102.0	4.4	101.1	6.7
	8:2 FTS		6:2 FTS ^13^C_2_ D_4_	Quantitative	10.94 ± 0.06	1.13 ± 0.00	2.02 ± 1.03	1.75 ± 0.07	IQL–250	0.9975	0.165	0.5	114.2	17.4	111.7	12.2
	5:3 FTC		PFBS ^13^C_4_	Quantitative	9.55 ± 0.11	1.22 ± 0.02	1.07 ± 0.18	1.09 ± 0.05	IQL–200	0.9959	0.033	0.1	74.4	22.1	76.4	23.8
	6:2 diPAP		Methyl Paraben ^13^C_6_	Quantitative	12.56 ± 0.17	1.61 ± 0.03	1.57 ± 0.34	1.56 ± 0.09	IQL–1000	0.9951	3.3	10	109.2	9.0	101.7	14.9
	8:2 diPAP*		Methyl Paraben ^13^C_6_	Semi-quantitative	13.81 ± 0.01	1.77 ± 0.01	1.99 ± 0.11	1.98 ± 0.047	IQL–100	0.9924	0.0033	0.01	149.1	11.5	147.6	25.7
Phosphonic/phosphinic acids	PFHxPA		PFOA ^13^C_8_	Quantitative	9.05 ± 0.02	0.93 ± 0.00	4.36 ± 1.21	4.13 ± 0.10	IQL–100	0.9973	0.033	0.1	95.3	20.9	92.9	23.4
	PFOPA		6:2 FTS ^13^C_2_ D_4_	Semi-quantitative	9.69 ± 0.03	1.00 ± 0.00	---	---	IQL–200	0.9988	0.165	0.5	97.5	7.0	104.3	24.2
	PFDPA		6:2 FTS ^13^C_2_ D_4_	Semi-quantitative	10.93 ± 0.03	1.13 ± 0.00	---	---	IQL–600	0.9961	0.165	0.5	91.8	20.3	89.1	24.8
	6:6 PFPiA		PFOA ^13^C_8_	Quantitative	11.83 ± 0.02	1.22 ± 0.00	---	6.47 ± 0.32	IQL–75	0.9917	0.165	0.5	101.3	29.5	88.3	22.7
	8:8 PFPiA*		PFOA ^13^C_8_	Semi-quantitative	13.13 ± 0.01	1.35 ± 0.00	---	---	IQL–750	0.9940	0.0165	0.05	51.7	28.8	46.9	28.2
	8:2 monoPAP*		6:2 FTS ^13^C_2_ D_4_	Semi-quantitative	10.80 ± 0.03	1.12 ± 0.00	14.07 ± 22.74	7.61 ± 0.54	IQL–50	0.9963	0.033	0.1	39.7	24.0	48.3	7.5
Novel emerging PFAS	Gen X		PFBS ^13^C_4_	Quantitative	8.57 ± 0.01	1.10 ± 0.00	1.88 ± 0.56	1.84 ± 0.06	IQL–200	0.9987	0.033	0.1	88.8	23.2	92.2	18.3
	ADONA		PFBS ^13^C_4_	Quantitative	9.06 ± 0.02	1.16 ± 0.00	2.58 ± 0.90	2.34 ± 0.15	IQL–250	0.9995	0.033	0.1	92.2	10.8	90.4	11.6
	9ClPF_3_ONS		Acetaminophen D_4_	Quantitative	10.55 ± 0.02	1.97 ± 0.01	69.59 ± 19.81	61.67 ± 6.05	IQL–1000	0.9962	8.25	25	55.6	9.2	56.6	9.6

Asterisk (*) denotes compounds that are classed as semi-quantitative as a result of having only one transition, or for poor accuracy or precision

### Method performance

Table 5 shows the parameters for method performance ordered by PFAS class. The method performance parameters were determined from spiked influent wastewater samples. Retention times did not change significantly in matrix, with all compounds eluting earlier in influent samples than in the mobile phase, except for PFOSA, 8:2 diPAP, and MeFOSA, which both eluted later in matrix samples. For HLB cartridges, the method detection limits (MDL) ranged from 0.03 (PFBS) to 756.9 ng L^−1^ (MeFOSA) and MQLs ranged from 0.06 to 2293.6 ng L^−1^ for the same compounds. This higher end of MDLs is driven by a high IQL for the sulfonamides and sulfonamido acetic acids in this method.

Recoveries were generally within the acceptable range (50–150%). Standard deviations are higher, as recovery was assessed at as many of the 4 spiked concentrations that fell within calibration range. This is more representative of PFAS matrix recovery given their presence at both trace levels, but also high levels when industry is a high contributor to the wastewater. At each concentration level, variation between replicate vials of same concentration was low. Poor recovery was seen for longer-chain PFAS such as PFUnDA, PFTrDA, and PFTeDA. Other hydrophobic PFAS such as 6:2 diPAP and 8:8 PFPiA showed high variability in their recovery across concentration levels. This aligns with a study looking at PFAS in bird and mammalian serum, where recoveries for diPAPs, PFPiAs, and long-chain PFAS were lower and more variable than others [63], as well as lower recoveries for sulfonamides and sulfonamido acetic acids [63]. This was seen also in another study, where it was suggested that concentrating the sample under nitrogen after elution could be a factor in this loss [70]. Lower method recovery could also be attributed to lack of available matching ISTDs, or to matrix effects. 8:2 monoPAP could not be recovered in wastewater, and other studies have found much lower recoveries for this compound relative to others in the method [72, 73].

Method accuracy and precision were calculated, with accuracies ranging from 32.36 to 167.85% with most compounds within the acceptable range for full quantification. Poorer accuracy and precision were seen for PFPeA, PFOSA, and EtFOSAA. PFBA has shown to act unexpectedly in matrix, with lower concentration level spiked matrix samples sometimes failing to reach the S/N threshold for quantification.

This method is therefore novel in that it spans a range of the most prevalent and used PFAS classes, with both novel emerging and legacy PFAS being quantified for in influent wastewater. Although there are several published methods also spanning a range of PFAS classes, this is the only method that includes this combination of PFAS, alongside the three novel PFAS: Gen X, ADONA, and 9ClPF_3_ONS to assess changes in PFAS usage over time. Method validation is comparable to other published methods, where matrix recovery, procedural blanks, and regular injection of reference standards are injected to further validate the method. Ion ratios used for full quantification are between ± 40%, as the recommendation by the published EPA method 1633 A is ± 50% [74]. Furthermore, method detection and quantification limits for certain compounds such as PFBS and PFBA are significantly lower in this method compared to others [46, 48, 49], allowing for more comprehensive trace analysis.

**Table 5:** Method performance parameters for PFAS in influent wastewater matrix

Analyte class	Analyte	Quantification	RT (mins)	RRT (mins)	Ion ratio	Method recovery (%)	MDL (ng L^−1^)	MQL (ng L^−1^)	Method accuracy (%)	Method precision (%)
PFSAs	PFBS	Quantitative	7.71 ± 0.02	1.00 ± 0.00	0.51 ± 0.17	118.2 ± 18.5	0.03	0.08	80.5	4.7
	PFHxS	Quantitative	8.99 ± 0.02	1.17 ± 0.00	1.70 ± 0.22	110.4 ± 18.3	0.3	0.9	91.0	8.9
	PFHpS	Semi-quantitative	9.64 ± 0.02	1.00 ± 0.00	40.47 ± 7.68	103.2 ± 13.2	159.9	484.6	86.2	11.5
	PFOS	Semi-quantitative	10.23 ± 0.08	1.06 ± 0.01	4.71 ± 1.25	170.8 ± 31.8	1.0	2.9	92.7	20.8
	PFNS	Quantitative	10.31 ± 0.00	1.10 ± 0.00	6.76 ± 2.61	80.9 ± 18.7	102.0	308.9	98.9	23.2
PFSA precursors	PFOSA	Semi-quantitative	11.73 ± 0.08	1.52 ± 0.01	41.71 ± 16.77	70.6 ± 27.0	467.2	1415.8	111.9	42.6
	n-MeFOSA	Semi-quantitative	12.68 ± 0.13	1.32 ± 0.01	2.26 ± 1.71	43.6 ± 0.6	756.9	2293.6	99.8	1.7
	n-EtFOSA	Quantitative	12.97 ± 0.09	1.35 ± 0.01	1.33 ± 1.14	33.2 ± 1.4	496.7	1505.1	94.0	2.5
	n-MeFOSAA	Quantitative	10.99 ± 0.03	1.08 ± 0.02	1.59 ± 0.99	44.4 ± 19.1	7.4	22.5	90.4	36.8
	n-EtFOSAA	Quantitative	11.26 ± 0.02	1.41 ± 0.01	5.14 ± 4.72	31.1 ± 13.8	106.2	321.9	136.3	32.0
PFCAs	PFBA	Quantitative	6.92 ± 0.04	0.98 ± 0.04	---	122.7 ± 31.5	0.03	0.08	90.4	15.4
	PFPeA	Quantitative	7.62 ± 0.02	0.75 ± 0.01	595.74 ± 376.73	104.0 ± 19.0	31.8	96.2	146.6	24.4
	PFHxA	Quantitative	8.32 ± 0.01	0.82 ± 0.01	14.43 ± 2.38	125.0 ± 28.3	1.3	4.0	123.8	25.6
	PFHpA	Quantitative	8.97 ± 0.02	0.88 ± 0.01	4.32 ± 0.60	128.3 ± 27.5	0.1	0.4	95.1	20.4
	PFOA	Quantitative	9.64 ± 0.02	1.00 ± 0.00	3.27 ± 0.42	113.0 ± 21.2	0.2	0.4	87.9	7.4
	PFNA	Quantitative	10.25 ± 0.02	1.06 ± 0.00	4.49 ± 0.67	149.5 ± 25.6	2.2	6.7	85.4	20.4
	PFDA	Quantitative	10.74 ± 0.02	1.11 ± 0.00	9.22 ± 6.72	103.2 ± 31.3	1.6	4.9	108.4	18.2
	PFUnDA	Quantitative	11.20 ± 0.02	1.16 ± 0.00	12.50 ± 24.11	50.1 ± 24.5	65.9	199.8	68.9	15.4
	PFDoDA	Quantitative	11.75 ± 0.02	1.22 ± 0.00	8.76 ± 4.21	44.0 ± 11.4	37.5	113.7	85.6	6.2
	PFTrDA	Semi-quantitative	12.30 ± 0.02	1.28 ± 0.00	1.46 ± 0.56	11.5 ± 5.2	14.3	43.3	68.9	29.6
	PFTeDA	Semi-quantitative	12.77 ± 0.02	1.60 ± 0.01	5.80 ± 1.02	7.6 ± 2.9	0.4	1.3	119.7	28.3
PFCA precursors	6:2 FTS	Quantitative	9.64 ± 0.02	1.00 ± 0.00	10.40 ± 2.43	110.4 ± 15.8	1.5	4.5	75.5	8.8
	8:2 FTS	Quantitative	10.33 ± 0.01	1.10 ± 0.00	1.85 ± 0.50	296.3 ± 61.9	0.6	1.7	59.0	9.1
	5:3 FTC	Quantitative	9.42 ± 0.02	1.22 ± 0.00	1.06 ± 0.20	107.6 ± 20.4	0.3	0.9	88.4	27.9
	6:2 diPAP	Quantitative	12.50 ± 0.03	1.62 ± 0.00	1.71 ± 0.33	20.8 ± 5.7	158.8	481.2	131.0	27.9
	8:2 diPAP	Semi-quantitative	14.94 ± 0.02	1.95 ± 0.09	2 ± 0.16	16.8 ± 6.1	0.2	0.6	129.0	18.1
	PFHxPA	Quantitative	8.98 ± 0.02	0.93 ± 0.00	5.22 ± 1.70	135.3 ± 15.5	0.2	0.7	91.7	26.2
Phosphonic/phosphinic acids	PFOPA	Semi-quantitative	9.52 ± 0.02	0.99 ± 0.00	---	107.6 ± 30.5	1.5	4.7	94.3	5.6
	PFDPA	Semi-quantitative	10.34 ± 0.02	1.10 ± 0.00	---	239.1 ± 73.5	0.7	2.1	86.1	14.0
	6:6 PFPiA	Quantitative	11.67 ± 0.02	1.21 ± 0.00	11.33 ± 5.90	45.5 ± 16.4	3.6	11.0	63.3	5.8
	8:8 PFPiA	Semi-quantitative	13.13 ± 0.02	1.36 ± 0.00	---	6.3 ± 1.7	2.6	8.0	114.3	25.5
	8:2 monoPAP	Semi-quantitative	10.71 ± 0.12	1.11 ± 0.01	---	---	---	---	---	---
Novel emerging PFAS	Gen X	Quantitative	8.49 ± 0.01	1.10 ± 0.00	1.84 ± 0.32	103.3 ± 18.8	0.3	1.0	71.4	16.7
	ADONA	Quantitative	8.99 ± 0.01	1.17 ± 0.00	2.26 ± 0.83	75.7 ± 12.2	0.4	1.3	69.7	13.8
	9ClPF_3_ONS	Quantitative	10.46 ± 0.01	1.96 ± 0.02	61.26 ± 5.60	114.0 ± 22.5	72.4	219.3	93.7	13.8

### Elution solvent strength in SPE

**Fig. 2:**
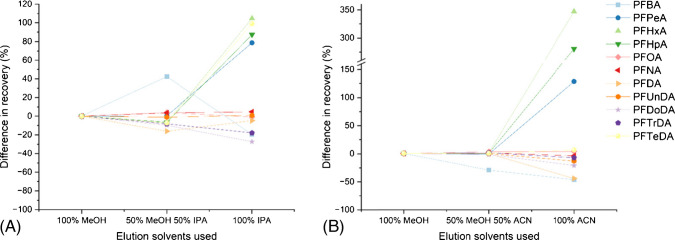
The difference in percentage recovery between 100% MeOH, 50% MeOH and 50% IPA, and 100% IPA (**A**), 100% MeOH, 50% MeOH and 50% ACN, and 100% ACN (**B**) for the class of carboxylic acids

**Fig. 3:**
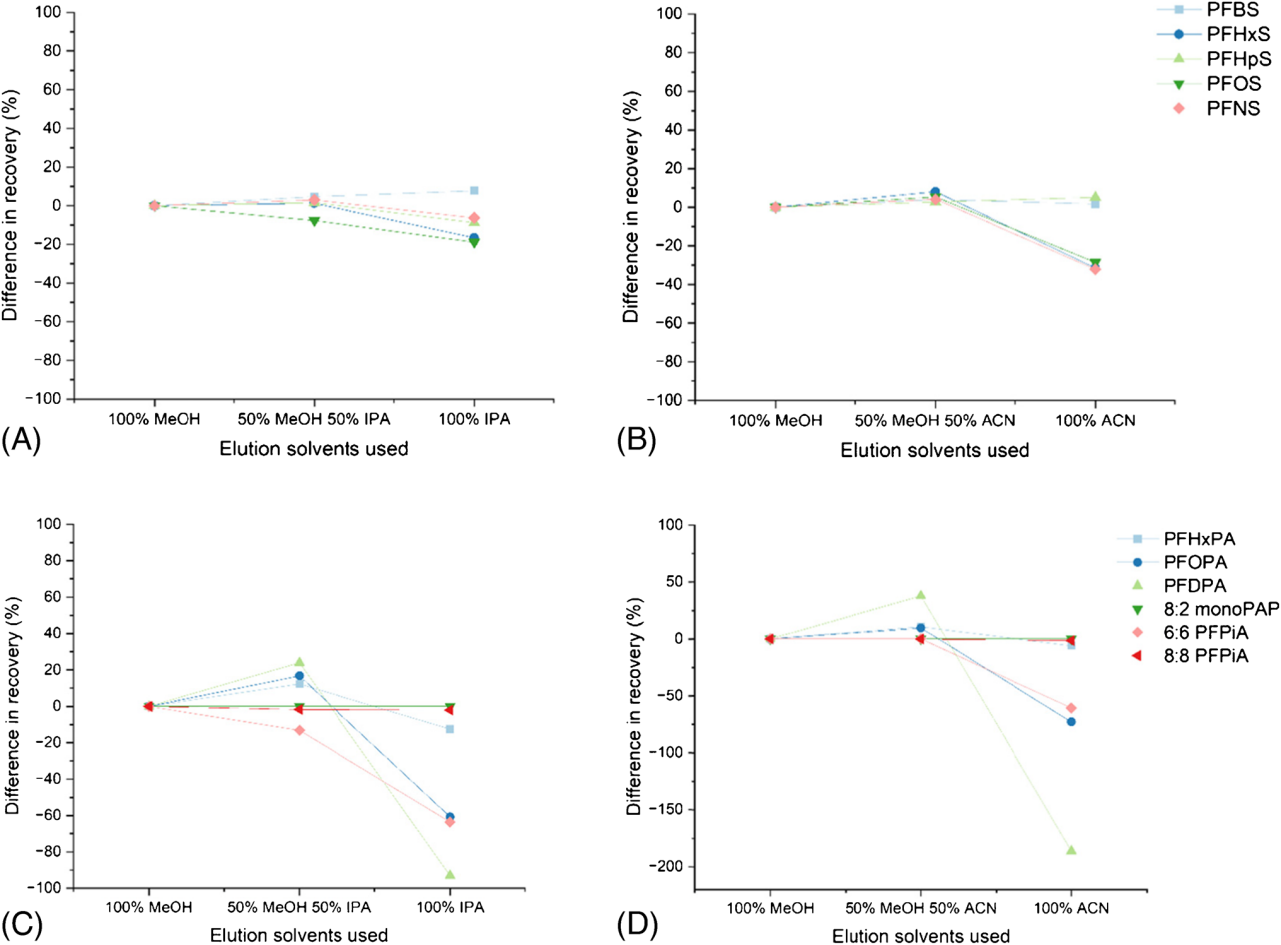
The difference in percentage recovery between 100% MeOH, 50% MeOH and 50% IPA, and 100% IPA (**A**), 100% MeOH, 50% MeOH and 50% ACN, and 100% ACN (**B**) for the class of sulfonic acids and for the class of phosphonic acids (**C**) and (**D**)

**Fig. 4:**
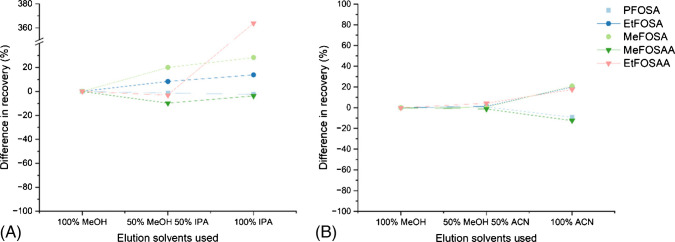
The percentage difference in recovery between 100% MeOH, 50% MeOH and 50% IPA, and 100% IPA (**A**), 100% MeOH, 50% MeOH and 50% ACN, and 100% ACN (**B**) for the class of sulfonic acid precursors

**Fig. 5:**
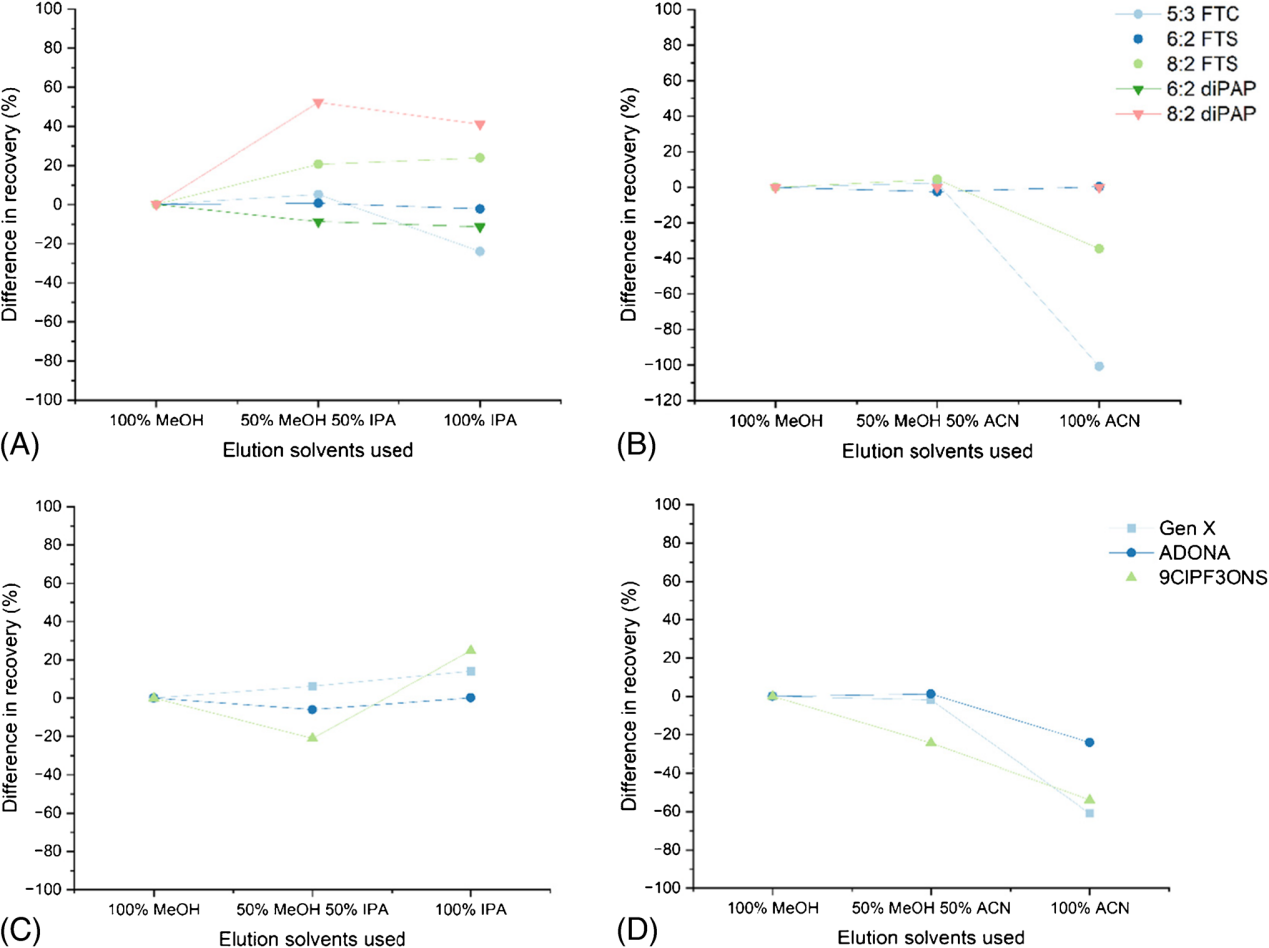
The percentage difference in recovery between 100% MeOH, 50% MeOH and 50% IPA, and 100% IPA (**A**), 100% MeOH, 50% MeOH and 50% ACN, and 100% ACN (**B**) for the class of carboxylic acid precursors, and the class of novel emerging PFAS (**C**) and (**D**)

Lower recoveries were seen for longer-chain PFAS on HLB cartridges. This could be due to a range of reasons, such as loss of long-chain PFAS to filter paper [75], suggesting that other methods, such as direct injection, could be more appropriate for these compounds, as has been previously done in environmental water samples [76]. This could also be from adsorption to solid particulate in the matrix [77]. Another possible reason is that compounds with a higher Log P could be adsorbing to the cartridge sorbent to a higher degree than those with a Log P and are not effectively eluted using MeOH. IPA and ACN were tested as alternative solvents, due to their higher degrees of hydrophobicity and higher solvent strength [78]. The results can be seen grouped by PFAS class in Figs. 2, 3, 4, and 5.

There were no significant improvements when using a stronger solvent for elution for the carboxylic acids. Four compounds, PFPeA, PFHxA, PFHpA, and PFTeDA, saw large increases in recovery using both IPA and ACN; however, this is not from enhanced signal of analyte; it is from their matching to a non-PFAS internal standard (Ibuprofen D_3_ for PFPeA, PFHxA, and PFHpA and Ketoprofen D_3_ for PFTeDA) which had a dramatically reduced signal when eluting with 100% stronger solvent, thus enhancing the analyte response. There were some marked reductions in recovery using 100% ACN for some PFAS such as PFBA (−46%), PFDA (−44%), PFUnDA (−14%), and PFDoDA (−21%), and in 100% IPA for PFBA (−19%), PFDoDA (−27%), and PFTrDA (−18%).

There were no significant improvements for the class of sulfonic acids; however, there were some reductions in recovery using 100% ACN for PFHxS (−31%), PFOS (−29%), and PFNS (−32%). For the phosphonic/phosphinic class of compounds, PFOPA and PFDPA saw a marked reduction in recovery for both IPA and ACN (−61% and −93%, respectively, for IPA, and −73% and −186%, respectively, for ACN). 6:6 PFPiA also saw a notable reduction using IPA and ACN (−64% and −61%, respectively). 8:2 monoPAP could not be recovered in this matrix using any of the three solvents.

There were some improvements in recovery for the sulfonic acid precursors using IPA and ACN. Both MeFOSA and EtFOSA had enhanced recoveries, with improvements of 28% and 14% for IPA, respectively, and improvements of 21% and 20% for ACN, respectively. EtFOSAA had enhanced recovery; however, similar to some of the carboxylic acids, this is from having a non-PFAS ISTD assigned to it (Ketoprofen D_3_), which behaves differently with the stronger solvents as opposed to higher peak area. For MeFOSAA and PFOSA, reductions in recovery are derived from both analyte and ISTD loss of peak area.

8:2 FTS showed an improvement (24%) using IPA. 8:2 diPAP had a significantly better recovery when eluting with IPA, resulting from an enhanced analyte and ISTD signal. With ACN, 8:2 diPAP and 6:2 diPAP showed high variability and could therefore not be conclusive, so have been set to 0 on the figure. 5:3 FTC showed a reduction in recovery when eluting with both IPA and ACN (−24% and −101%, respectively). The novel PFAS showed slight improvements in recovery with IPA; however, they all saw a decrease in recovery using ACN.

Overall, these results show that for this method that spans a range of PFAS from short chain to long chain with varying functional groups, MeOH remains the best solvent for their extraction in one method. However, if a method targeting perfluorinated sulfonamide compounds, 8:2 FTS or 8:2 diPAP, was developed, IPA or ACN would be more appropriate solvents and may explain their poorer recovery in this method compared to other compounds. Perfluorinated sulfonamides (FASAs) can exist as neutral compounds, with a higher degree of hydrophobicity than other PFAS of the same chain length [79], with FASA chain length driving this [80].

ACN generally performed worse than both IPA and MeOH, which could be because it does not have the polar hydroxy group, or because it has been suggested in the literature previously that PFAS may be unstable in ACN and other aprotic solvents, with Gen X performing the worst in this solvent [81], also backed by this study. Another study also suggested that MeOH works as a better elution solvent than ACN [68].

### PFAS adsorbance to silanised glassware and HDPE bottles

During sample preparation, spiked wastewater is left to equilibrate in HDPE bottles for 30–60 min. Furthermore, eluted compounds are dried under nitrogen in silanised tubes for further reconstitution in the appropriate solvent composition. However, it has been noted in the literature that PFAS do adsorb to glass and plastic [82, 83], with hydrophobicity being a significant driver of this [84]; therefore, the extent to which this occurs in this method was evaluated to address the lower method recovery and instrumental accuracies seen for some compounds.

Accuracy and precision in the mobile phase were assessed by comparing mobile phase QCs prepared as normal, ‘dry down’; by spiking analyte directly into the PP vial, ‘in vial’; and left in an HDPE bottle for 40 min, ‘plastic adsorbance’. This was assessed at one concentration level per sample, either 200 ng mL^−1^ or 20 ng mL^−1^, dependent on calibration range, to remove any variation from different concentration ranges when comparing. To ensure that there was no leaching from the PP vials used for instrumental analysis, blanks of 80:20 H_2_O:MeOH were looked at, with no PFAS concentrations present.

**Fig. 6:**
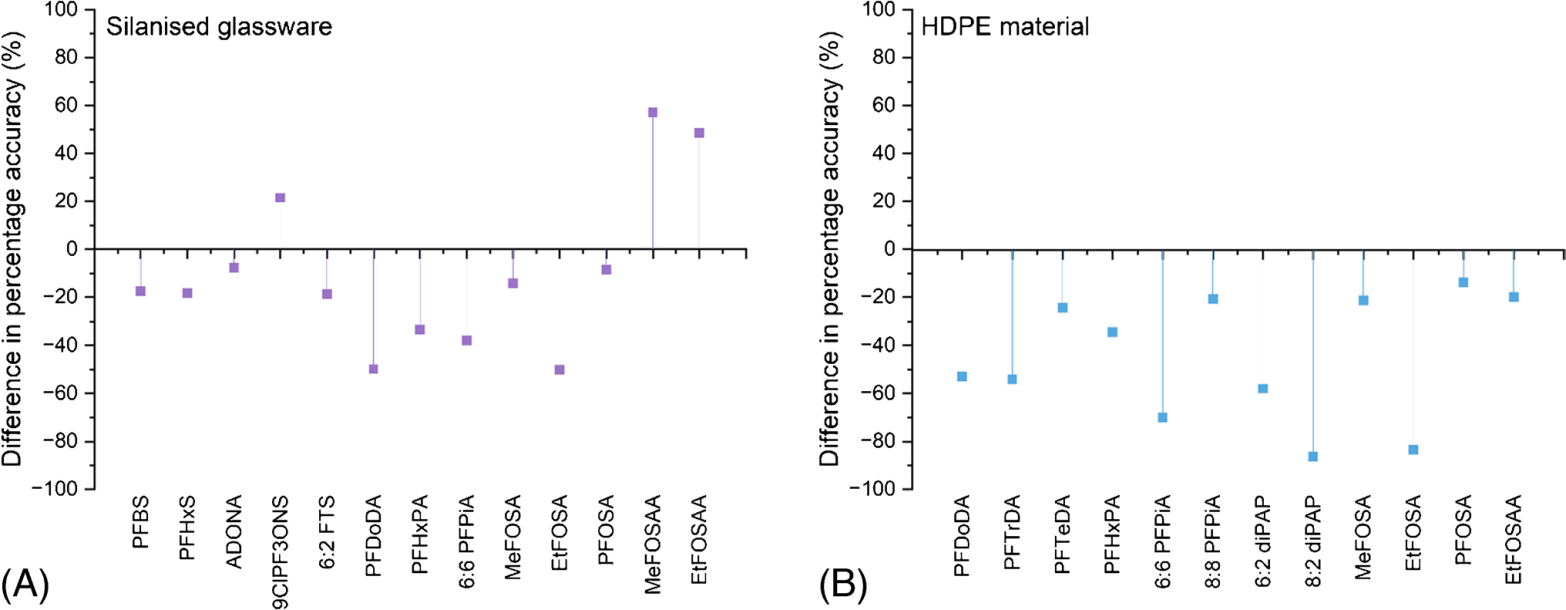
The change in percentage accuracy when samples are dried in silanised tubes ‘dry down’ (**A**) and when left in a HDPE bottle for 40 min ‘plastic adsorbance’ (**B**)

Generally, there were decreases in the accuracy of ‘dry down’ and ‘plastic adsorbance’ samples compared to ‘in vial’, with most compounds. However, these compounds had this reduction within the standard deviation of the accuracy of the tested samples or the in-vial sample; therefore, these will not be reported. Other compounds, however, saw a significant reduction that fell safely outside the standard deviations of both samples, suggesting a real difference in concentration. Results are shown in Fig. 6 and Table 6, and log *P* values can be found in S2. For Table 6, the suitability of material was assessed, with suitable (< 15%), medium suitability (15% ≤ × < 30%), and potentially unsuitable (≥ 30%). Full accuracy and precision can be found in S8, showing the standard deviations of these percentage changes.

**Table 6.**
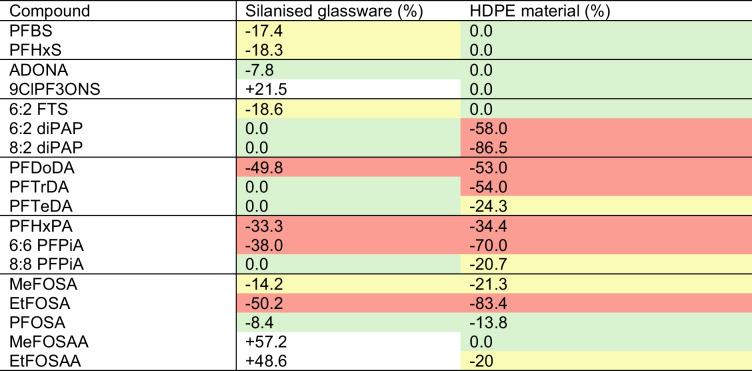
Suitability of silanised glassware and HDPE material based on loss of analyte

The results firstly for drying down show the difference in accuracy, ranging from percentage reductions ranging from 7.8% (ADONA) to 50.2% (EtFOSA). The long-chain carboxylic acid PFDoDA is affected by this sample preparation step. FASAs all showed worse accuracy when drying down, with the extent of this being more pronounced with EtFOSA, as it has a higher Log *P* value. Interestingly, however, the perfluorinated sulfonamido acetic acids and 9ClPF_3_ONS showed a better accuracy with drying down. This could possibly be explained by where Zenobio et al. noted a change in adsorbance level when analytes were spiked in separately or as a mixture, suggesting that competitive adsorption or selective adsorption may occur [84]. It is therefore possible that the adsorption of another PFAS leads to an enhanced signal of another.

In terms of PFAS plastic adsorbance, percentage reductions ranged from 13.8% (PFOSA) to 86.5% (8:2 diPAP). More PFAS showed a lower accuracy in plastic adsorbance compared to dry down, suggesting that plastic has a negative effect on more PFAS than glass, as has been suggested by Cao et al. [83]. Pairs of phosphorous-based PFAS of similar chemistries such as the PFPiAs and diPAPs showed adsorbance to HDPE. Both FASAs in this method adsorbed to the plastic, as has been seen previously [84], which again is the result of their ability to exist as neutral species. The sulfonamido acetic acid EtFOSAA had a reduction in accuracy of 50.5%; however, the reduction for MeFOSAA fell within the standard deviation of the samples and therefore cannot reliably be attributed to adsorption. This may be from EtFOSAA’s higher Log P value than MeFOSAA.

### Assessment of public exposure

**Fig. 7:**
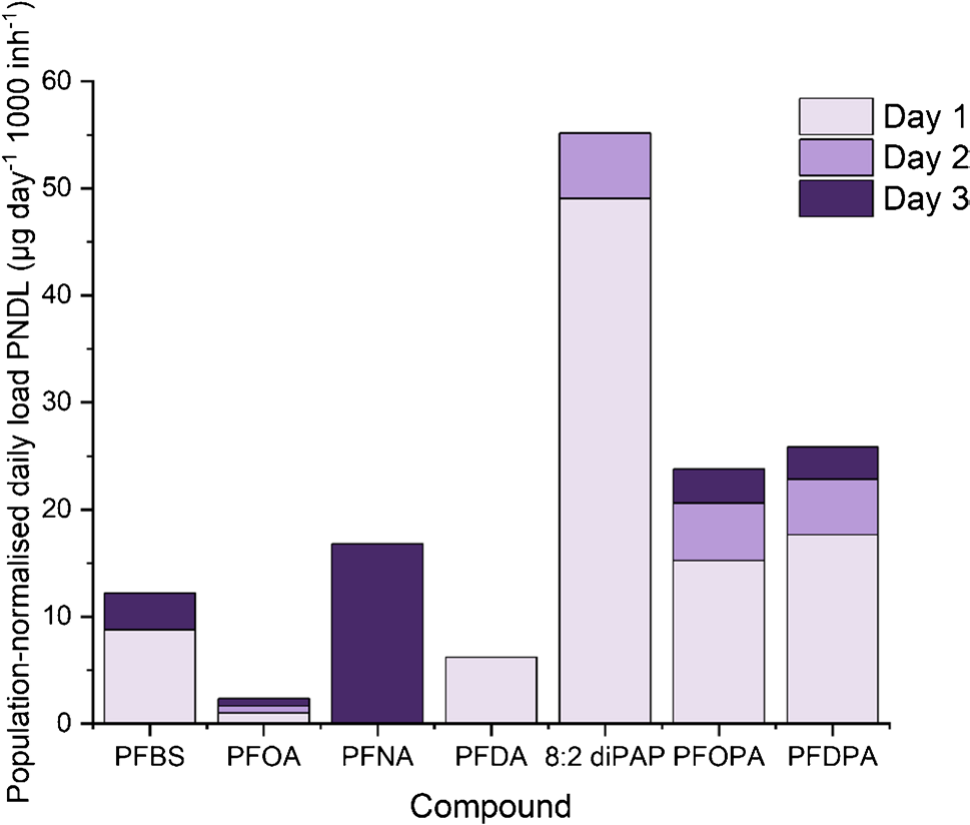
A bar chart showing the population-normalised daily loads (µg day^−1^ 1000 inh^−1^) of the quantifiable PFAS in these 3-day samples

This method was applied to 3 consecutive days of influent wastewater samples from the South-West of England, in February. The results for this can be seen below in Table 7 and Fig. 7. PFAS across 4 classes were quantifiable, with 3 of those being semi-quantifiable (PFBS, PFOPA, PFDPA) as one transition is reported, and 4 fully quantifiable (PFOA, PFNA, PFDA, 8:2 diPAP). The highest cumulative concentration was seen for 8:2 diPAP at 198.1 ± 109.1 ng L^−1^, and the lowest recorded concentration (> LOQ) was seen for PFOA at 8.5 ± 0.6 ng L^−1^. Daily loads ranged from 1935 ± 147 mg day^−1 ^(PFOA) to 45,369 ± 24,984 mg day^−1^ (8:2 diPAP). PNDL ranged from 2.5 ± 0.2 µg day^−1^ 1000 inh^−1^ (PFOA) to 55.2 ± 30.4 µg day^−1^ 1000 inh^−1^ (8:2 diPAP).

PNDI (population-normalised daily intake) cannot be calculated for the assessment of PFAS exposure, resulting from the lack of a characteristic human metabolite for each compound, resulting from their reluctance to break down and long half-lives [85–87], highlighting the need for progression in this field. Monitoring these compounds in wastewater still presents an opportunity to understand risk to human health, as it can be assumed that a proportion of the PFAS load is derived from exposure. There is uncertainty around the best approach for PFAS TDIs (tolerable daily intake) given their complexity and assumptions that have to be taken [88] as well as continuing research on their negative health effects [89–91]. The EFSA has set a TWI (tolerable weekly intake) of 4.4 ng/kg bw [92]. Presenting a ‘worst-case’ scenario where PNDLs here are directly translated to a TWI by this definition, the TWI for the highest PNDL presented here for 8:2 diPAP (55.2 ± 30.4 µg day^−1^ 1000 inh^−1^) is 1.61 ng/kg bw for men and 1.90 ng/kg bw for women, showing that levels of potential exposure are safely below recommended limits. Due to PFAS bioaccumulation, wastewater levels should be looked at alongside levels seen in HBM for a comprehensive understanding of exposure.

Table 8 compares PFAS concentrations in this study to other PFAS studies of influent wastewater. S9 shows the values of DL and PNDL also. Levels of phosphonic acids have not been extensively studied globally, with levels in this study higher than in other studies. Concentrations of PFBS, PFOA, PFDA, and PFNA were comparable to other similar studies and fell within ranges previously reported. DLs and PNDLs were significantly higher than those reported in Italy [93] and significantly lower than those reported in Romania [94]. Novel PFAS Gen X, ADONA, and 9ClPF3ONS were not detected in this study, despite low MDLs for Gen X and ADONA. 9ClPF3ONS has a high MDL driven by a high IQL in this method which could be responsible for its non-detection. 9ClPF3ONS has been detected frequently in China, corresponding to discharge from the semiconductor industry [95]. However, it has also been detected at levels of up to 0.08 ng L^−1^ in the UK Thames River, as well as Gen X which was detected at up to 1.58 ng L^−1^ [96].

**Table 7:** Concentrations, daily loads, and population-normalised daily loads of the full target list of compounds in three consecutive days of influent wastewater

Analyte class	Analyte name	Detection Frequency *n* = *3*	Day 1 (ng L^−1^)	Day 2 (ng L^−1^)	Day 3 (ng L^−1^)		Cumulative concentration (ng L^−1^)	Cumulative daily load (DL) (mg day^−1^)	Cumulative population-normalised daily load (PNDL) (µg day^−1^ 1000 inh^−1^)
PFSAs	PFBS	2	31.5**	< LOD	12.4**	43.8 ± 13.5		10,039 ± 3092	12 ± 3
	PFHxS	0	< LOD	< LOD	< LOD	0		0	0
	PFHpS	0	< LOD	< LOD	< LOD	0		0	0
	PFOS	0	< LOD	< LOD	< LOD	0		0	0
	PFNS	0	< LOD	< LOD	< LOD	0		0	0
PFSA Precursors	PFOSA	0	< LOD	< LOD	< LOD	0		0	0
	n-MeFOSA	0	< LOD	< LOD	< LOD	0		0	0
	n-EtFOSA	0	< LOD	< LOD	< LOD	0		0	0
	n-MeFOSAA	0	< LOD	< LOD	< LOD	0		0	0
	n-EtFOSAA	0	< LOD	< LOD	< LOD	0		0	0
PFCAs	PFBA	1	< LOQ	< LOD	< LOD	0		0	0
	PFPeA	0	< LOD	< LOD	< LOD	0		0	0
	PFHxA	0	< LOD	< LOD	< LOD	0		0	0
	PFHpA	1	< LOQ	< LOD	< LOD	0		0	0
	PFOA	3	3.6	2.4	2.5**	8.5 ± 0.6		1935 ± 147	4 ± 0.2
	PFNA	1	< LOD	< LOD	60.4	20.1 ± 0.00		13,825 ± 0	17 ± 0
	PFDA	1	22.3	< LOD	< LOD	22.3 ± 0.00		5099 ± 0	6 ± 0
	PFUnDA	0	< LOD	< LOD	< LOD	0		0	0
	PFDoDA	0	< LOD	< LOD	< LOD	0		0	0
	PFTrDA*	0	< LOD	< LOD	< LOD	0		0	0
	PFTeDA*	0	< LOD	< LOD	< LOD	0		0	0
PFCA Precursors	6:2 FTS	1	< LOD	< LOD	< LOQ	0		0	0
	8:2 FTS	0	< LOD	< LOD	< LOD	0		0	0
	5:3 FTC	0	< LOD	< LOD	< LOD	0		0	0
	6:2 diPAP	0	< LOD	< LOD	< LOD	0		0	0
	8:2 diPAP	3	176.2	21.9	< LOQ	198.1 ± 109.1		45,369 ± 24,984	55 ± 30
Phosphonic/phosphinic acids	PFHxPA	0	< LOD	< LOD	< LOD	0		0	0
	PFOPA	3	54.8*	19.3*	11.4*	85.4 ± 23.1		19,563 ± 5296	24 ± 6
	PFDPA	3	63.4*	18.6*	10.9*	92.9 ± 28.4		21,280 ± 6500	26 ± 8
	6:6 PFPiA	0	< LOD	< LOD	< LOD	0		0	0
	8:8 PFPiA*	0	< LOD	< LOD	< LOD	0		0	0
	8:2 monoPAP*	0	< LOD	< LOD	< LOD	0		0	0
Novel emerging PFAS	Gen X	0	< LOD	< LOD	< LOD	0		0	0
	ADONA	0	< LOD	< LOD	< LOD	0		0	0
	9ClPF_3_ONS	0	< LOD	< LOD	< LOD	0		0	0

Asterisk (*) denotes semi-quantification resulting from only one transition in the method. Asterisks (**) denote semi-quantification resulting from one transition being reported here

**Table 8:** A comparison of levels of PFAS found in this study with similar studies globally

Study location (No. of influent sampling points)	Most prevalent PFAS	PFBS (ng L^−1^)	PFOA (ng L^−1^)	PFDA (ng L^−1^)	PFNA (ng L^−1^)	8:2 diPAP (ng L^−1^)	PFOPA (ng L^−1^)	PFDPA (ng L^−1^)
This study	PFBS, PFOA, PFDA, PFNA, 8:2 diPAP, PFOPA, PFDPA	12.4–31.5	2.4–3.6	22.3	60.4	21.9–176.2	11.4–54.8	10.9–63.4
Belgium [20, 48]	PFBS, PFOS, PFBA, PFPeA, PFOA, PFDA, 8:2 FTS	2.5–23.0	2.4–2726.0	< 0.1–1.9	0.2–8.6	N/A	< LOD	< LOD
Sweden [49]	6:2 FTS, PFHxS, PFBA, PFHxA	4.1–4.9	4.6–8.9	< LOD	< LOD–0.25	N/A	N/A	N/A
Sweden [3, 72]	PFCAs, PFBA, PFHxA, PFOS, PFBS,	0.6–3.2	2.8–5.1	< LOQ–0.4	0.2–0.7	< LOQ–2.9	1.8–5.0	< LOQ–4.4
Spain [32, 97]	PFBA, PFNA, PFPeA, PFOS, PFBS	0.02–305.0	0.04–107.0	0.04–128	0.06–178.0	N/A	N/A	N/A
Italy [16, 93]	PFPeA, MeFOSAA, PFOS, PFBS, PFHxS	1.2–6.5	1.0–7.6	< LOQ–1830	0.02–1.7	N/A	N/A	N/A
Romania [5, 94]	PFOA, PFOS, PFPeA, PFHxA, PFDA, PFUnDA	N/A	227–318	15.1–21.2	N/A	N/A	N/A	N/A
Canada [27, 47]	6:2 FTS, 5:3 FTC, PFBA, PFPeA, PFHxA, PFOS, PFOA	2.0–68.0	1.1–146.0	< LOQ–33	N/A	N/A	N/A	N/A
Australia [18, 45]	PFHxA, PFOS, 6:2 FTS	2.3 ± 0.71–20 ± 18	3.2 ± 1.4–13 ± 17	< LOQ–3.6 ± 18	N/A	N/A	N/A	N/A
Korea [15, 50]	PFHxA, PFOA, PFBS, PFOS	7.4 ± 6–110 ± 150	30 ± 56–550 ± 1000	3.3 ± 5.7–27 ± 48	N/A	N/A	N/A	N/A

### Assessment of environmental risk

Table 9 shows an assessment of the environmental risk from PFAS concentrations. As only influent wastewater was processed for this study, assessment of environmental risk can be looked at in the event of a combined sewer overflow. This would present the worst-case scenario, especially as the dilution factor into rivers can vary. A dilution factor of tenfold is often assumed [98], which would significantly lessen the risk, suggesting that risk to the receiving environment in this site is fairly low; however, different countries have had different dilutions predicted for them [99].

PNEC values taken from the Norman database are evaluated by either experimental ecotoxicology data (where possible) or Quantitative Structure - Activity Relationship (QSAR) reports [60]. Values were taken for freshwater. The risk quotients were calculated and assessed as low risk (< 0.1), medium risk (0.1 ≤ RQ < 1), and high risk (≥ 1). The results show a high risk for 8:2 diPAP (2.4) on day 1 and a medium risk on day 2 (0.30). Medium risk was also seen for PFDA (0.13) and PFDPA (0.22).

The high and medium risks were calculated for acute toxicity in fish; however, chronic exposure should be taken into account to ensure that bioaccumulation potential and toxicity over time are integrated into the calculation [100, 101].

**Table 9.**
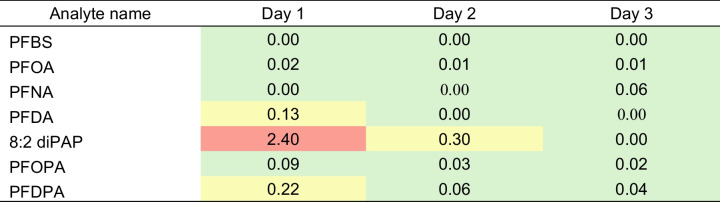
Environmental risk assessment using the risk quotient method for the quantifiable PFAS in this study

## Conclusions

A UPLC-MS/MS method was developed to allow for a comprehensive understanding of the use and exposure to 35 PFAS compounds in wastewater. This method comprised perfluoroalkyl carboxylic acids and their precursors, perfluoroalkyl sulfonic acids and their precursors, perfluorophosphonic acids, perfluorophosphinic acids, and novel/emerging ethers. The method also includes 4 isotopically labelled PFAS standards used as internal standards. Overall, accuracy and precision were within acceptable levels, apart from a few longer-chain compounds which showed low accuracy and high variability across concentrations. PFCAs and PFSAs worked best in this method, with less ideal parameters seen for PFSA precursor compounds. Recovery was acceptable for most compounds, with high recovery seen for compounds with matching ISTDs. Recovery was lower for more hydrophobic PFAS, with more variation seen across concentration levels as well.

An evaluation of the effectiveness of SPE cartridges was carried out, with WAX cartridges across two pH ranges, and HLB cartridges compared against each other. WAX cartridges showed potentially better suitability for shorter chain compounds, and HLB showed better suitability across the range of classes in this method.

IPA and ACN were tested as alternative eluents to MeOH to enhance the recovery of hydrophobic PFAS in HLB cartridges. MeOH was found to be suitable for the range of compounds in this method; however, both IPA and ACN improved the recoveries of FASAs MeFOSA and EtFOSA. IPA acted as a better eluent for 8:2 diPAP also. ACN generally performed worse than both MeOH and IPA.

The extent of PFAS adsorbance during sample preparation was evaluated, with adsorbance to HDPE material affecting PFAS accuracies to a higher extent than silanised glassware. Sulfonic acid precursor compounds were seen to adsorb more to these laboratory materials than other PFAS, as well as PFHxPA and some long-chain PFAAs. This addresses some of the key questions that are faced during PFAS sample preparation and analysis.

This method was applied to influent wastewater samples over 3 days where 10 PFAS were detected. Quantifiable PFAS were PFBS, PFOA, PFDA, PFNA, 8:2 diPAP, PFOPA, and PFDPA, with concentrations in line with those reported in literature previously. There was no detection of novel emerging PFAS. Levels of PFAS were safely below TWI recommendations; however, more information on human metabolism and integration of information on the bioaccumulative potential of PFAS and impact of chronic exposure, as well as combined PFAS effects, is needed to properly assess risk. Environmental risk to the surrounding environment was likely not significant, with potential risk arising from levels of 8:2 diPAP. More understanding of the level of PFAS adsorption to solids is needed to avoid underestimation of risk.

## Supplementary Information

Below is the link to the electronic supplementary material.

Supplementary file1 (DOCX 227 KB)

## Data Availability

All data are included in the paper.
