# Supplementary material for: A new multi-residue method for PFAS analysis in wastewater for environmental and public health risk assessment
Source: Anal Bioanal Chem. 2026 Mar 12;418(9):2917–38. doi: 10.1007/s00216-026-06421-5 (PMC13079494; doi:10.1007/s00216-026-06421-5)
Supplement: Supplementary file 1 — Supplementary file1 (DOCX 227 KB) [file 216_2026_6421_MOESM1_ESM.docx]

**Electronic Supplementary Material:**

**A new multi-residue method for PFAS analysis in wastewater for environmental and public health risk assessment**

Dalia Elabbadi^a, b, c^, Harry Elliss^a, b, c^, Megan Robertson^b, d^, John Bagnall^b, d^, Barbara Kasprzyk-Hordern^a, b, c^

^a^ Department of Chemistry, University of Bath, Claverton Down, Bath, BA2 7AY, UK

^b^ Centre of Excellence in Water-Based Early-Warning Systems for Health Protection, University of Bath, Claverton Down, Bath, BA2 7AY, UK

^c^ Institute of Sustainability and Climate Change, University of Bath, Claverton Down, Bath, BA2 7AY, UK

^d^ Wessex Water Service Ltd, Claverton Down, Bath BA2 7WW, UK

Corresponding Author

* Corresponding author at: Department of Chemistry, University of Bath, Bath BA2 7AY, UK. Email: Bkh20@bath.ac.uk

Author Email Addresses:

[Dmhe20@bath.ac.uk](mailto:Dmhe20@bath.ac.uk)

[Hjce20@bath.ac.uk](mailto:Hjce20@bath.ac.uk)

[Megan.robertson@wessexwater.co.uk](mailto:Megan.robertson@wessexwater.co.uk)

John.Bagnall@wessexwater.co.uk

[Bkh20@bath.ac.uk](mailto:Bkh20@bath.ac.uk)

**S1**: Purchasing Information of all analytes and internal standards

| Compound | Full name | Supplier |
| --- | --- | --- |
| PFBS | Perfluorobutane sulfonic acid | Merck |
| PFHxS | Perfluorohexane sulfonic acid | LGC standards |
| PFHpS | Perfluoroheptane sulfonic acid | Merck |
| PFOS | Perfluorooctane sulfonic acid | Merck |
| PFNS | Perfluorononane sulfonic acid | LGC standards |
| PFOSA | Perfluorooctanesulfonamide | LGC standards |
| n-MeFOSA | Methyl perfluorooctane sulfonamide | LGC standards |
| n-EtFOSA | Ethyl perfluorooctane sulfonamide | LGC standards |
| n-MeFOSAA | Methyl perfluorooctane sulfonamidoacetic acid | LGC standards |
| n-EtFOSAA | Ethyl perfluorooctane sulfonamidoacetic acid | LGC standards |
| PFBA | Perfluorobutanoic acid | Merck |
| PFPeA | Perfluoropentanoic acid | Merck |
| PFHxA | Perfluorohexanoic acid | Merck |
| PFHpA | Perfluoroheptanoic acid | Merck |
| PFOA | Perfluorooctanoic acid | Merck |
| PFNA | Perfluorononanoic acid | Merck |
| PFDA | Perfluorodecanoic acid | Merck |
| PFUnDA | Perfluoroundecanoic acid | Merck |
| PFDoDA | Perfluorododecanoic acid | Merck |
| PFTrDA | Perfluorotridecanoic acid | Merck |
| PFTeDA | Perfluorotetradecanoic acid | Merck |
| 6:2 FTS | 6:2-fluorotelomersulfonic acid | LGC standards |
| 8:2 FTS | 8:2-fluorotelomersulfonic acid | LGC standards |
| 5:3 FTC | 5:3 Fluorotelomer carboxylic acid | LGC standards |
| 6:2 diPAP | Bis[2-(perfluorohexyl)ethyl] phosphate | LGC standards |
| 8:2 diPAP | Bis[2-(perfluorooctyl)ethyl] phosphate | LGC standards |
| PFHxPA | Perfluorohexyl phosphonic acid | LGC standards |
| PFOPA | Perfluorooctyl phosphonic acid | LGC standards |
| PFDPA | Perfluorodecyl phosphonic acid | LGC standards |
| 6:6 PFPiA | Bis (perfluorohexyl) phosphinic acid | LGC standards |
| 8:8 PFPiA | Bis (perfluorooctyl) phosphinic acid | LGC standards |
| 8:2 monoPAP | Mono[2-(perfluorooctyl)ethyl] phosphate | LGC standards |
| Gen X | 2,3,3,3-tetrafluoro-2-(1,1,2,2,3,3,3-heptafluoropropoxy) propanoic acid | LGC standards |
| ADONA | 2,2,3-trifluoro-3-[1,1,2,2,3,3-hexafluoro-3-(trifluoromethoxy)propoxy]propanoic acid | LGC standards |
| 9ClPF_3_ONS | 6:2 chlorinated polyfluoroalkyl ether sulfonate | LGC standards |

**S2:** Full target list of PFAS in this method

| Compound Class | Compound | Full name | CAS number | Molecular formula | Molecular weight (g mol^-1^) | LogP | pKa |
| --- | --- | --- | --- | --- | --- | --- | --- |
| Sulfonic acids | PFBS | Perfluorobutane sulfonic acid | 375-73-5 | C_4_HF_9_O_3_S | 300.10 | 2.3 | -3.31 |
|  | PFHxS | Perfluorohexane sulfonic acid | 355-46-4 | C_6_HF_13_O_3_S | 400.12 | 3.7 | -3.32 |
|  | PFHpS | Perfluoroheptane sulfonic acid | 3871-99-6 | C_7_HF_15_O_3_S | 450.12 | 4.3 | -3.32 |
|  | PFOS | Perfluorooctane sulfonic acid | 2795-39-3 | C_8_HF_17_O_3_S | 500.13 | 5 | -3.32 |
|  | PFNS | Perfluorononanesulfonic acid | 68259-12-1 | [C9HF19O3S](https://pubchem.ncbi.nlm.nih.gov/#query=C9HF19O3S) | 550.14 | 5.7 | -3.34 |
| Precursors to sulfonic acids | PFOSA | Perfluorooctanesulfonamide | 754-91-6 | C_8_H_2_F_17_NO_2_S | 499.15 | 4.8 | 3.37 |
|  | n-Me-FOSA | Methyl perfluorooctane sulfonamide | 31506-32-8 | C_9_H_4_F_17_NO_2_S | 513.17 | 5.2 |  |
|  | n-Et-FOSA | Ethyl perfluorooctane sulfonamide | 4151-50-2 | C_10_H_6_F_17_NO_2_S | 527.20 | 5.6 |  |
|  | n-MeFOSAA | Methyl perfluorooctane sulfonamidoacetic acid | 2355-31-9 | C_11_H_6_F_17_NO_4_S | 571.21 | 5 | 2.5 |
|  | n-Et-FOSAA | Ethyl perfluorooctane sulfonamidoacetic acid | 2991-50-6 | C_12_H_8_F_17_NO_4_S | 585.24 | 5.4 | 2.5 |
| Carboxylic acids | PFBA | Perfluorobutanoic acid | 375-22-4 | C_4_HF_7_O_2_ | 214.04 | 2.2 | 0.37 |
|  | PFPeA | Perfluoropentanoic acid | 2706-90-3 | C_5_HF_9_O_2_ | 264.05 | 2.9 | 0.34 |
|  | PFHxA | Perfluorohexanoic acid | 307-24-4 | C_6_HF_11_O_2_ | 314.05 | 3.6 | 0.32 |
|  | PFHpA | Perfluoroheptanoic acid | 375-85-9 | C_7_HF_13_O_2_ | 364.06 | 4.3 | 0.31 |
|  | PFOA | Perfluorooctanoic acid | 335-67-1 | C_8_HF_15_O_2_ | 414.07 | 4.9 | 0.3 |
|  | PFNA | Perfluorononanoic acid | 375-95-1 | C_9_HF_17_O_2_ | 464.08 | 5.6 |  |
|  | PFDA | Perfluorodecanoic acid | 335-76-2 | C_10_HF_19_O_2_ | 514.08 | 6.3 | 0.4 |
|  | PFUnDA | Perfluoroundecanoic acid | 2058-94-8 | C_11_HF_21_O_2_ | 564.09 | 6.9 | 0.4 |
|  | PFDoDA | Perfluorododecanoic acid | 307-55-1 | C_12_HF_23_O_2_ | 614.10 | 7.6 | 0.4 |
|  | PFTrDA | Perfluorotridecanoic acid | 72629-94-8 | C_13_HF_25_O_2_ | 664.10 | 8.3 | 0.4 |
|  | PFTeDA | Perfluorotetradecanoic acid | 376-06-7 | C_14_HF_27_O_2_ | 714.11 | 9 | 0.4 |
| Precursors to carboxylic acids | 6:2 FTS | 6:2-fluorotelomersulfonic acid | 27619-97-2 | C_8_H_5_F_13_O_3_S | 428.17 | 3.9 |  |
|  | 8:2 FTS | 8:2-fluorotelomersulfonic acid | 39108-34-4 | C_10_H_5_F_17_O_3_S | 528.18 | 5.3 |  |
|  | 5:3 FTC | 5:3 Fluorotelomer carboxylic acid | 914637-49-3 | C_8_H_5_F_11_O_2_ | 342.11 | 3.9 |  |
|  | 6:2 diPAP | Bis[2-(perfluorohexyl)ethyl] Phosphate | [57677-95-9](https://commonchemistry.cas.org/detail?cas_rn=57677-95-9) | [C16H9F26O4P](https://pubchem.ncbi.nlm.nih.gov/#query=C16H9F26O4P) | 790.17 | 8.6 |  |
|  | 8:2 diPAP | Bis[2-(perfluorooctyl)ethyl] phosphate | 678-41-1 | C_20_H_9_F_34_O_4_P | 990.2 | 11.3 |  |
| Phosphorous-based compounds | PFHxPA | Perfluorohexyl phosphonic acid | 40143-76-8 | C_6_H_2_F_13_O_3_P | 400.03 | 3 | 0.46 |
|  | PFOPA | Perfluorooctyl phosphonic acid | 40143-78-0 | C_8_H_2_F_17_O_3_P | 500.05 | 4.3 | 0.46 |
|  | PFDPA | Perfluorodecyl phosphonic acid | 52299-26-0 | C_10_H_2_F_21_O_3_P | 600.06 | 5.6 | 0.46 |
|  | 6:6 PFPiA | Bis (perfluorohexyl) phosphinic acid | 40143-77-9 | C_12_HF_26_O_2_P | 702.07 | 8 |  |
|  | \| 8:8 PFPiA \| Bis(heptadecafluorooctyl)phosphinic acid \| 40143-79-1 \| [C16HF34O2P](https://pubchem.ncbi.nlm.nih.gov/#query=C16HF34O2P) \| 902.1 \| \| --- \| --- \| --- \| --- \| --- \| | Bis(heptadecafluorooctyl)phosphinic acid | 40143-79-1 | [C16HF34O2P](https://pubchem.ncbi.nlm.nih.gov/#query=C16HF34O2P) | 902.1 | 10.7 |  |
|  | 8:2 MonoPAP | Mono[2-(perfluorooctyl)ethyl] phosphate | 57678-03-2 | C_10_H_6_F_17_O_4_P | 544.10 | 4.6 |  |
| Novel/ Emerging | Gen X | 2,3,3,3-tetrafluoro-2-(1,1,2,2,3,3,3-heptafluoropropoxy) propanoic acid | 13252-13-6 | C_6_HF_11_O_3_ | 330.05 | 3.6 |  |
|  | ADONA | 2,2,3-trifluoro-3-[1,1,2,2,3,3-hexafluoro-3-(trifluoromethoxy)propoxy]propanoic acid | 919005-14-4 | C_7_H_2_F_12_O_4_ | 378.07 | 4.1 |  |
|  | 9ClPF_3_ONS | 6:2 chlorinated polyfluoroalkyl ether sulfonate | 73606-19-6 | C_8_ClF_16_KO_4_S | 570.67 | n/a |  |

Log P Computed by XLogP3 3.0 (PubChem release 2025.09.15)

pKa calculated using Chemicalize 2022, where available


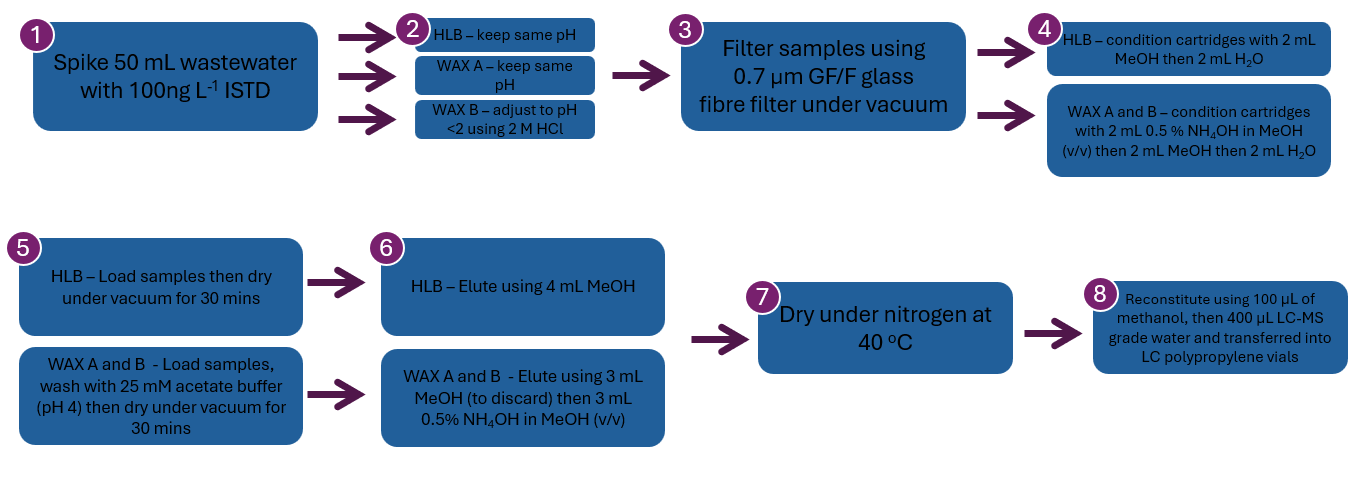
**S3:** Schematic of HLB vs WAX cartridge sample preparation

**S4:** Flow and population data for WBE samples.

| **Day** | **Flow (L/day)** | **Population (inhabitants)** |
| --- | --- | --- |
| 1 | 229043117 | 822456 |
| 2 | 215157945.6 | 822456 |
| 3 | 184854096 | 822456 |

**S5.** TIC of all compounds in this method


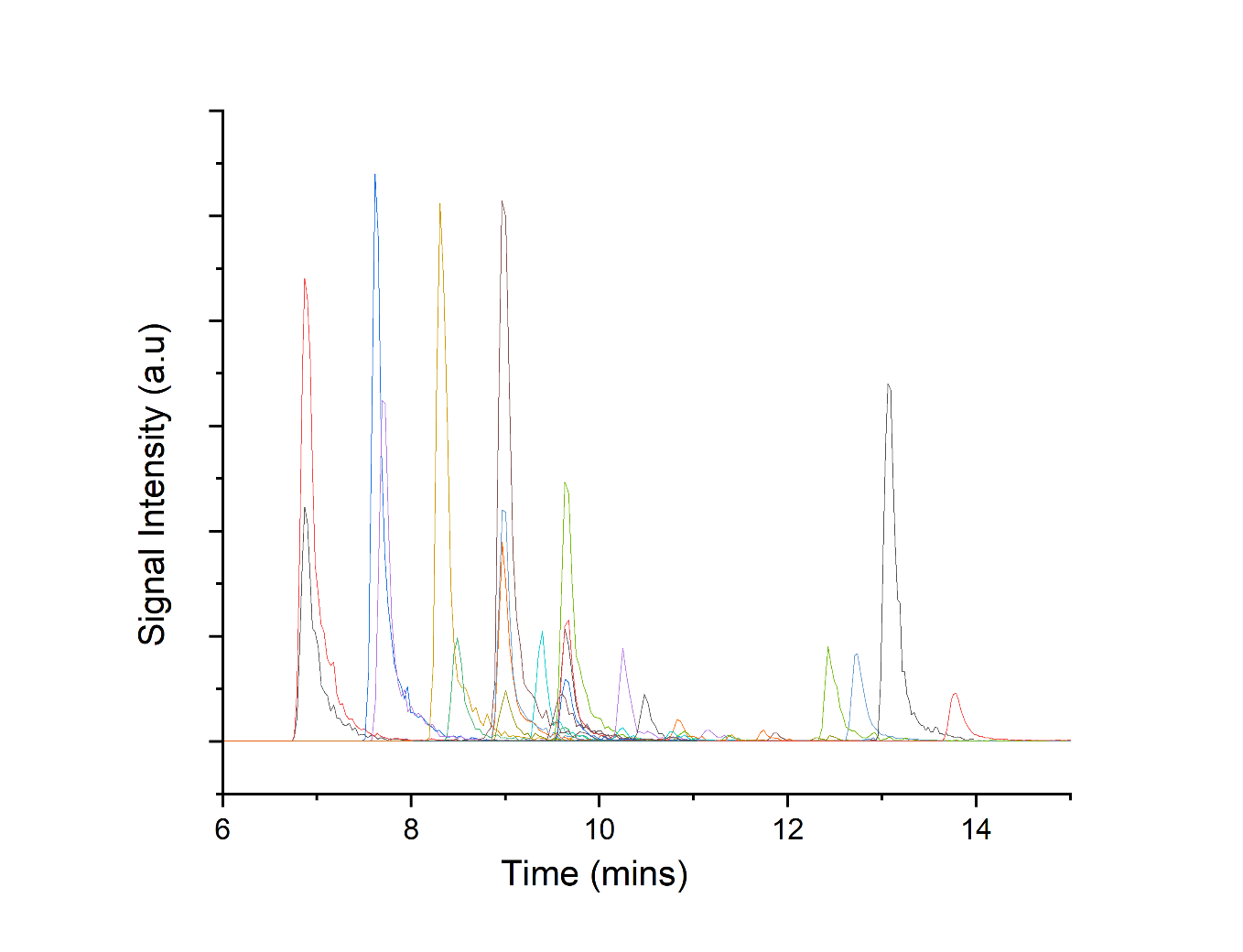


**S6**: Method recovery for WAX vs HLB at 200 or 20 ng mL^-1^. *denotes those at which recovery was calculated at 20 ng mL^-1^.

|  | HLB A | ST.DEV | WAX A | ST.DEV | HLB B | ST.DEV | WAX B | ST.DEV |
| --- | --- | --- | --- | --- | --- | --- | --- | --- |
| PFBA | 137.31 | 25.46 | 104.52 | 6.16 | 107.78 | 9.43 | 90.88 | 2.77 |
| PFPeA | 178.29 | 11.77 | 155.65 | 39.94 | 87.76 | 10.57 | 124.59 | 33.09 |
| PFBS | 126.23 | 5.47 | 98.08 | 10.88 | 210.71 | 6.85 | 95.48 | 3.42 |
| PFHxA | 100.42 | 6.68 | 130.31 | 29.72 | 176.30 | 11.38 | 162.44 | 41.22 |
| Gen X | 97.10 | 3.25 | 65.83 | 12.55 | 107.41 | 7.49 | 37.01 | 8.24 |
| PFHpA | 137.73 | 10.34 | 146.52 | 36.38 | 176.37 | 11.38 | 110.97 | 28.01 |
| PFHxS | 97.97 | 2.71 | 92.95 | 14.10 | 107.75 | 11.80 | 43.91 | 9.91 |
| PFHxPA* | 114.53 | 12.42 | 98.61 | 7.55 | 137.12 | 13.02 | 311.76 | 41.93 |
| ADONA | 61.85 | 3.87 | 58.49 | 10.75 | 66.01 | 6.62 | 24.27 | 5.79 |
| 5:3 FTC | 98.63 | 5.50 |  |  | 130.51 | 14.79 | 0.00 | 0.00 |
| 9ClPF_3_ONS | 126.95 | 8.53 | 164.38 | 29.30 | 114.03 | 22.53 | 18.92 | 3.74 |
| PFOPA | 133.15 | 4.96 | 119.21 | 12.78 | 105.47 | 7.85 | 4.64 | 1.48 |
| PFHpS | 105.50 | 5.55 | 111.34 | 12.09 | 101.65 | 11.87 | 37.58 | 8.90 |
| PFOA | 104.71 | 7.74 | 90.21 | 6.03 | 97.16 | 5.34 | 81.77 | 2.47 |
| 6:2 FTS | 98.95 | 3.93 | 92.46 | 8.63 | 99.06 | 6.69 | 88.37 | 4.31 |
| PFOS | 152.49 | 6.52 | 201.46 | 26.39 | 168.22 | 31.92 | 13.65 | 4.49 |
| PFNA | 153.43 | 10.18 | 156.22 | 7.02 | 136.09 | 20.95 | 22.52 | 5.16 |
| 8:2 monoPAP | |  |  |  |  |  |  |  |
| PFNS | 56.94 | 6.27 | 1.52 | 0.46 | 73.75 | 17.16 | 17.87 | 1.64 |
| PFDPA | 238.35 | 10.97 | 140.75 | 22.72 | 246.23 | 21.99 | 0.00 | 0.00 |
| PFDA | 84.03 | 3.06 | 149.46 | 19.62 | 95.26 | 19.26 | 8.34 | 2.19 |
| 8:2 FTS | 206.66 | 13.00 | 253.28 | 15.45 | 279.59 | 52.75 | 12.42 | 4.48 |
| MeFOSAA |  |  |  |  | 32.17 | 6.78 | 1.61 | 0.30 |
| PFUnDA | 22.03 | 2.57 | 23.53 | 7.48 | 27.40 | 4.92 | 5.75 | 0.51 |
| EtFOSAA |  |  |  |  | 25.39 | 7.80 | 0.76 | 0.04 |
| PFOSA | 91.49 | 0.72 | 89.08 | 0.06 | 106.93 | 0.67 | 0.00 | 0.00 |
| 6:6 PFPiA* | 24.71 | 6.06 | 27.67 | 11.65 | 50.11 | 20.59 | 6.10 | 0.76 |
| PFDoDA* | 31.21 | 5.01 | 20.67 | 15.15 | 43.99 | 11.44 | 24.97 | 0.88 |
| PFTrDA* | 7.69 | 2.64 | 2.25 | 1.87 | 10.73 | 4.77 | 0.00 | 0.00 |
| MeFOSA |  |  |  |  | 43.60 | 0.64 |  |  |
| 6:2 diPAP | 19.51 | 10.06 | 18.94 | 15.62 | 25.59 | 2.66 | 19.80 | 0.33 |
| EtFOSA | 33.41 | 2.20 |  |  | 33.22 | 1.41 | 30.71 | 0.24 |
| PFTeDA | 6.48 | 2.82 |  |  | 4.40 | 1.00 | 2.54 | 0.08 |
| 8:8 PFPiA | 6.93 | 0.08 | 11.02 | 0.08 | 11.25 | 0.42 | 8.44 | 0.45 |
| 8:2 diPAP* | 16.75 | 6.11 | 6.93 | 0.08 | 18.77 | 5.68 |  |  |

**S7:** Further discussion on signal intensities for HLB vs WAX cartridges

Looking at signal intensities at the same spiked level as in the figure below, WAX (A) cartridges were found to have higher signal intensities than HLB cartridges, with the exception seen for 5:3 FTC, PFOSA, EtFOSA, EtFOSAA, PFTrDA, PFTeDA, 8:8 PFPiA, MeFOSA, 6:2 diPAP, 8:2 diPAP. Except for 5:3 FTC, these were the compounds with higher retention times. Certain compounds had higher intensities with WAX cartridges by an order of magnitude 10. These were PFBA, PFHxPA, 6:2 FTS, PFDA and 8:2 monoPAP as illustrated by Figure S1 below for PFBA. On WAX (B) cartridges, HLB had a higher signal intensity for all compounds except for PFBA.


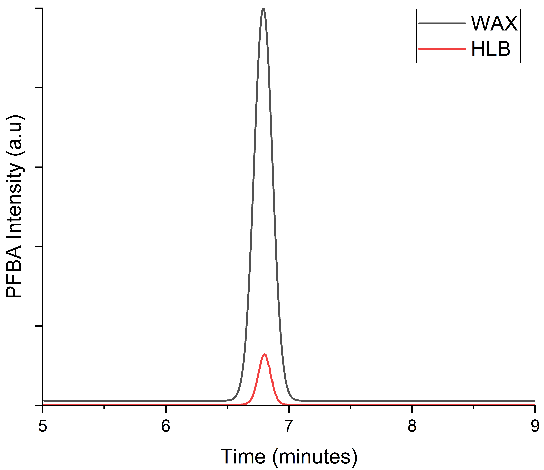


**Fig S1.**  Signal Intensity for PFBA on HLB (red) against WAX A (grey)

Looking at the signal intensities of the PFAS ISTDs used in this method, higher signal intensities were seen on using WAX (A) for all ISTDs, but PFBS ^13^C_4_, which showed similar signal intensities. Using WAX (B), PFBA ^13^C_4_ and PFBS ^13^C_4_ both had higher signal intensities compared to HLB. This, alongside the method recovery suggests better extraction for PFBS using WAX (B).

**S8:** Accuracies and precision for the adsorbance tests

| Compound class | Compound | ‘in vial’ accuracy | ‘in vial’ precision | ‘dry down’ accuracy | ‘dry down’ precision | ‘adsorbance’ accuracy | ‘adsorbance’ precision |
| --- | --- | --- | --- | --- | --- | --- | --- |
| Sulfonic Acids | PFBS | 124.9 | 1.4 | 107.6 | 4.4 | 122.8 | 4.3 |
|  | PFHxS | 108.9 | 5.8 | 90.6 | 3.8 | 107.2 | 7.8 |
|  | PFHpS | 147.9 | 7.2 | 127.1 | 15.7 | 165.0 | 13.4 |
|  | PFOS | 118.5 | 6.0 | 115.3 | 20.6 | 177.5 | 41.9 |
|  | PFNS | 142.9 | 23.2 | 127.6 | 24.8 | 179.2 | 26.9 |
| Precursors to sulfonic acids | PFOSA | 113.1 | 1.7 | 104.7 | 1.7 | 99.3 | 4.8 |
|  | MeFOSA | 127.9 | 0.2 | 113.7 | 3.1 | 106.7 | 0.3 |
|  | EtFOSA | 114.2 | 15.6 | 64.0 | 17.6 | 30.8 | 0.4 |
|  | MeFOSAA | 34.8 | 2.5 | 92.1 | 11.8 | 31.4 | 8.8 |
|  | EtFOSAA | 39.6 | 4.7 | 88.2 | 20.6 | 19.6 | 8.1 |
| Carboxylic acids | PFBA | -- | -- | -- | -- | -- | -- |
|  | PFPeA | 94.7 | 6.05 | 91.4 | 11.3 | 106.8 | 4.2 |
|  | PFHxA | 104.3 | 9.3 | 128.0 | 6.7 | 121.6 | 7.6 |
|  | PFHpA | 80.7 | 4.2 | 106.8 | 16.9 | 91.4 | 4.4 |
|  | PFOA | 96.6 | 3.0 | 86.5 | 6.8 | 92.7 | 2.4 |
|  | PFNA | 79.0 | 8.0 | 75.4 | 8.2 | 91.2 | 7.7 |
|  | PFDA | 77.4 | 8.0 | 79.3 | 13.9 | 95.7 | 14.3 |
|  | PFUnDA | 110.7 | 6.3 | 99.0 | 17.8 | 107.1 | 16.7 |
|  | PFDoDA | 116.5 | 15.1 | 66.7 | 9.9 | 64.2 | 23.8 |
|  | PFTrDA | 59.9 | 33.6 | 23.9 | 9.2 | 5.9 | 4.5 |
|  | PFTeDA | 25.6 | 4.9 | 26.7 | 8.0 | 1.3 | 0.5 |
| Precursors to carboxylic acids | 6:2 FTS | 106.5 | 1.7 | 87.8 | 7.4 | 104.3 | 3.5 |
|  | 8:2 FTS | 134.3 | 15.8 | 127.5 | 26.2 | 182.1 | 36.0 |
|  | 5:3 FTC | 78.5 | 13.5 | 115.22 | 25.5 | 103.3 | 22.6 |
|  | 6:2 diPAP | 87.2 | 5.2 | 89.0 | 46.4 | 29.3 | 2.7 |
|  | 8:2 diPAP | 111.7 | 17.2 | 107.4 | 37.4 | 25.3 | 7.1 |
| Phosphorous based acids | PFHxPA | 112.7 | 4.2 | 79.5 | 4.7 | 78.4 | 3.9 |
|  | PFOPA | 116.2 | 8.0 | 108.6 | 4.8 | 108.3 | 6.1 |
|  | PFDPA | 83.8 | 7.4 | 150.5 | 43.9 | 83.1 | 10.9 |
|  | 6:6 PFPiA | 94.0 | 10.6 | 55.9 | 6.6 | 24.0 | 7.7 |
|  | 8:8 PFPiA | 27.5 | 2.4 | 30.5 | 18.8 | 6..8 | 0.0 |
|  | 8:2 monoPAP | 147.2 | 79.0 | 87.9 | 24.8 | 104.3 | 31.3 |
| Novel/emerging | Gen X | 147.1 | 6.9 | 126.5 | 6.0 | 150.1 | 6.6 |
|  | ADONA | 90.8 | 2.6 | 83.0 | 1.1 | 92.4 | 3.8 |
|  | 9ClPF3ONS | 51.2 | 5.1 | 72.6 | 7.4 | 80.9 | 18.8 |

| **S9**: A comparison of PFAS levels found in this study, with other studies of influent wastewater globally, including daily load and population-normalised daily load calculations.  Concentrations in ng L^-1^  Daily Load in mg day^-1^  Population-normalised Daily Load in µg day^-1^ 1000 inh^-1^  *A comparison of levels of PFAS found in this study with other studies of influent wastewater globally.* | | | | | | | | | | | | | | | | | | | | | | | |
| --- | --- | --- | --- | --- | --- | --- | --- | --- | --- | --- | --- | --- | --- | --- | --- | --- | --- | --- | --- | --- | --- | --- | --- |
| **Study location (No of influent sampling points)** | **Most prevalent PFAS** |  | **PFBS** | | **PFOA** | | | **PFDA** | | | | **PFNA** | | | **8:2 diPAP** | | | **PFOPA** | | | **PFDPA** | | |
|  |  | C | DL | PNDL | C | DL | PNDL | C | DL | PNDL | C | | DL | PNDL | C | DL | PNDL | C | DL | PNDL | C | DL | PNDL |
| This study | PFBS, PFOA, PFDA, PFNA, 8:2 diPAP, PFOPA, PFDPA | 12.4 – 31.5 | 2833 – 7205 | 3 – 9 | 2.4 – 3. | 541 – 813 | 0.7 – 1 | 22.3 | <LOQ - 5098 | <LOQ – 6 | 60.4 | | <LOQ - 13825 | <LOQ – 17 | 21.9 – 176.2 | 5018 - 40351 | 6 – 49 | 11.4 – 54.8 | 2604 - 12546 | 3 – 15 | 10.9 – 63.4 | 2497 - 14530 | 3 – 18 |
| Belgium (20)(48) | PFBS, PFOS, PFBA, PFPeA, PFOA, PFDA, 8:2 FTS | 2.5 – 23.0 |  |  | 2.4 – 2726.0 |  |  | <LOQ – 1.9 |  |  | 0.2 – 8.6 | |  |  | N/A |  |  | <LOD |  |  | <LOD |  |  |
| Sweden(49) | 6:2 FTS, PFHxS, PFBA, PFHxA | 4.1 – 4.9 | 84 - 101 |  | 4.6 – 8.9 | 95 - 183 |  | <LOD | <LOD |  | <LOD – 0.25 | | <LOD – 5.2 |  | N/A | N/A | N/A | N/A | N/A | N/A | N/A | N/A | N/A |
| Sweden (3)(72) | PFCAs, PFBA, PFHxA, PFOS, PFBS, | 0.6 – 3.2 |  |  | 2.8 – 5.1 |  |  | <LOQ – 0.4 |  |  | 0.2 – 0.7 | |  |  | <LOQ – 2.9 |  |  | 1.8 – 5.0 |  |  | <LOQ – 4.4 |  |  |
| Spain (32) (94) | PFBA, PFNA, PFPeA, PFOS, PFBS | 0.02 – 305.0 |  |  | 0.04 – 107.0 |  |  | 0.04 - 128 |  |  | 0.06 – 178.0 | |  |  | N/A |  |  | N/A |  |  | N/A |  |  |
| Italy (16)(92) | PFPeA, MeFOSAA, PFOS, PFBS, PFHxS | 1.2 – 6.5 | 0.65 – 1665 |  | 1.0 – 7.6 | 29.2 – 459 |  | <LOQ – 1830 | 0.11 – 138 |  | 0.02 – 1.7 | | 0.13 – 168 |  | N/A |  |  | N/A |  |  | N/A |  |  |
| Romania (5)(93) | PFOA, PFOS, PFPeA, PFHxA, PFDA, PFUnDA | N/A | N/A | N/A | 227.0 – 318.0 | 14,169 – 433,710 | 109,000 – 237,000 | 15.1 – 21.2 | 947 – 30012 | 67300 - 16400 | 3.2 – 4.0 | | 144 – 7155 | 1600 - 3910 | N/A |  |  | N/A |  |  | N/A |  |  |
| Australia(45) (18) | PFHxA, PFOS, 6:2 FTS. | 2.3 ± 0.71 – 20 ± 18 |  |  | 3.2 ± 1.4 – 13 ± 17 |  |  | <LOQ - 3.6 ± 18 |  |  |  | |  |  | N/A |  |  | N/A |  |  | N/A |  |  |
| Korea(50) (15) | PFHxA, PFOA, PFBS, PFOS | 7.4 ± 6 – 110 ± 150 |  |  | 30 ± 56 – 550 ± 1000 |  |  | 3.3 ± 5.7 – 27 ± 48 |  |  |  | |  |  | N/A |  |  | N/A |  |  | N/A |  |  |
| N/A refers to compounds that were not in the targeted list for that study | | | | | | | | | | | | | | | | | | | | | | | |
